# WNT5B regulates myogenesis and fiber type conversion by affecting mRNA stability

**DOI:** 10.7150/ijbs.102309

**Published:** 2025-06-09

**Authors:** Danyang Fan, Yilong Yao, Chao Yan, Fanqinyu Li, Yalan Yang, Bingkun Xie, Zhonglin Tang

**Affiliations:** 1 Shenzhen Branch, Guangdong Laboratory for Lingnan Modern Agriculture, Agricultural Genomics Institute at Shenzhen, Chinese Academy of Agricultural Sciences, Shenzhen 518124, China.; 2 Kunpeng Institute of Modern Agriculture at Foshan, Agricultural Genomics Institute, Chinese Academy of Agricultural Sciences, Foshan 528226, China.; 3 Key Laboratory of Livestock and Poultry Multi-Omics of MARA, Agricultural Genomics Institute at Shenzhen, Chinese Academy of Agricultural Sciences, Shenzhen 518124, China.; 4 Guangxi Key Laboratory of Livestock Genetic Improvement, Guangxi Institute of Animal Sciences, Nanning 530001, China.

**Keywords:** *WNT5B*, single nucleotide polymorphisms, mRNA stability, myogenesis, fiber type conversion

## Abstract

The wingless-integrated (WNT) signaling pathway is known to play a critical role in myogenesis. *WNT5B*, a member of the WNT family, is essential for determining cell fate and development. However, the molecular mechanisms by which *WNT5B* regulates myogenesis remain unclear. This study observed that *WNT5B*, which is conserved between mice and pigs, is highly expressed in the skeletal muscle. The expression of *WNT5B* was varied in the skeletal muscle between the Tongcheng (obese-type) and Landrace (lean-type) pigs. *In vitro*, *WNT5B* promoted skeletal muscle cell proliferation and cell cycle progression, while inhibited cell apoptosis. *In vivo*, *WNT5B* promoted myofiber thickness and increased slow-type muscle fibers in porcine skeletal muscles. Mechanically, a SNP site (c.1608 A > G) located in the 3' untranslated region (3'UTR) of *WNT5B* regulated transcript attenuation and acts through AU-rich element mediation (ARE) to influence myogenesis. Also, the SNP was located in the miRNA response element of miR-29a/b/c to influence *WNT5B* expression. However, ARE sites did not affect the binding relationship of miR-29a/b/c. In conclusion, this study demonstrated that the SNP (c.1608 A > G) affects *WNT5B* mRNA stability by protecting the 3'UTR of the *WNT5B* from the degrading effects of miR-29a/b/c. This study reveals the critical role of *WNT5B* in myogenesis and indicates that it is a novel candidate gene for pig breeding.

## Introduction

Skeletal muscle, which constitute the largest metabolic and endocytic organ system in the body, plays a crucial role in maintaining physiological characteristics and homeostasis 1, 2. The growth and development of skeletal muscle are highly precise, tightly coordinated, and well-organized multistep biological processes 3, involving myoblast proliferation 4, 5, differentiation 6, 7, and apoptosis 8, 9. Skeletal muscle growth and development are regulated by multiple signaling pathways. Among the key signaling pathways that orchestrate skeletal muscle development, the WNT signaling pathway has emerged as a critical regulator due to its involvement in cell fate determination, tissue patterning, and myogenic progression 10-12. However, the role of the WNT pathway, a profound signaling pathway 13, in skeletal muscle growth and development is not fully understood.

*WNT5B* (WNT Family Member 5B) is classified as a non-canonical WNT ligand and mainly functions through the non-canonical WNT pathway 14-16. It plays a key role in the development and physiological processes of various tissues, including bone 17, 18, adipose 19, and heart 20. Previous studies have demonstrated that *WNT5B* affects cell proliferation and migration and is involved in cancer and vascular smooth muscle cell differentiation 21, 22. Also, *WNT5B* regulate tissue polarity and cell migration 23, 24, and *WNT5B*-Ror2 complexes form in the producing cell and are handed over from these cytonemes to the receiving cell 25. Most studies on WNT5B have centered on its expression patterns and links to disease. However, the role of *WNT5B* in myogenesis is not well understood.

*WNT5B* is also a pivotal regulatory factor in mammalian health and disease and is regulated by genetic and epigenetic factor 26, 27. The SNP (rs2887571) at the *WNT5B* enhancer alters ERα binding, affecting WNT5B expression in osteoporosis 28. DNA methylation 29, 30, histone modification factors 31, 32, and non-coding RNAs (ncRNAs) 33, 34 have been shown to regulate *WNT5B* expression. The binding of ncRNA *tsRMST* to the PRC2 component SUZ12 inhibits *WNT5B* expression during human embryonic stem cell differentiation 35. Moreover, miR-5587-3P, miR-587, and miR-149-5P have been found to modulate *WNT5B* expression by binding to its 3' untranslated region (3'UTR) in, respectively, cancer, fat and chondrocytes cells 36-38. However, the specific regulatory mechanisms of *WNT5B* during myogenesis require further investigation.

In this study, the expression profiles of *WNT5B* were comparatively analyzed in different pig breeds at different developmental stages to investigate the regulatory role of *WNT5B* in myogenesis. A functional SNP site (c.1608 A > G) was identified and located in the 3'UTR of *WNT5B*, which regulates myogenesis through genetic and epigenetic mechanisms. Our findings suggest that *WNT5B* is a promising candidate gene for the regulation of myogenesis and has potential benefits in improving skeletal muscle health and animal breeding.

## Materials and Methods

### Samples collection

A total of 200 sows were included in this study. DNA samples were obtained from obese-type breeds (Tongcheng sows, n = 40; Bama sows, n = 35; Luchuan sows, n = 25) and lean-type breeds (Landrace sows, n = 100), the age of sows selected are all in 180 days.

### *WNT5B*-related Gene Set Enrichment Analysis (GSEA)

To explore the expression and enrichment of *WNT5B*, we employed an RNA-seq dataset spanning 27 developmental time points ranging from embryonic day 33 to postnatal day 180 in Tongcheng and Landrace pigs (NCBI: GSE157045). The WNT family genes were shown by heatmap via R “heatmap” package. In addition, GSEA-Gene Ontology (Gene Ontology) and Kyoto Encyclopedia of Genes and Genomes (GSEA-KEGG) analyses were performed by ranking the correlations between *WNT5B* and all genes and calculating the enrichment of the set of genes 39. GSEA was performed using the “ClusterProfiler” package in R (v4.2.3).

### Isolation and culture of porcine primary myoblasts

Porcine primary myoblasts were isolated from the biceps femoris of piglets that were less than one week of age. They have a good proliferation rate and myogenic stem cell properties, and are widely used in molecular and cellular experimental studies* in vitro* 7, 40. The skeletal muscle tissue was minced and digested using type II collagenase (300 U/mL; Gibco, USA) in an oscillating water bath at 37°C for 30 min. The digestion process was terminated by adding a high-glucose medium (DMEM; Gibco, USA) supplemented with 10% fetal bovine serum (FBS; Gibco, USA) and 2% double antibody. The mixture was then filtered through 40, 70, and 100 μm filters to remove tissue debris. The resulting cell mass was resuspended and cultured in RPMI-1640 medium. Purified satellite cells were transferred to plates coated with Matrigel (BD Biosciences, USA) for proliferation. When the porcine primary myoblasts reached 90% confluence, differentiation was induced by adding 5% horse serum (HS, Gibco, USA) to the DMEM.

### Cell culture and transfection

For the *in vitro* experiments, C2C12 myoblasts and HEK293T cells were obtained from the American Type Culture Collection. Porcine skeletal muscle cells were obtained from Guangzhou Xinyuan Technology Co. Ltd., China. C2C12 myoblasts and HEK293T cells were cultured in Dulbecco's Modified Eagle's medium (DMEM; Sigma, USA) supplemented with 10% fetal bovine serum (Atlanta Biologicals, China), 1% penicillin, 100 mg/mL streptomycin, and 1% glutamine (Mediatech, USA). The cells were incubated at 37℃ with 5% CO_2_. Plasmids and oligonucleotides were transfected into cells using Lipofectamine 3000 according to the manufacturer's instructions (Thermo Fisher, USA). Typically, transfection was performed when the cells reached 50-60% confluence.

### SNP sequencing

The 3'UTR regions of Tongcheng, Bama, Luchuan and Landrace porcine *WNT5B* were amplified by PCR using genomic DNA as a template. The amplified regions were sequenced using Sanger sequencing (Sangon Biotech, China).

### Vectors construction and oligonucleotides

The plasmids of pcDNA3.1 and pcDNA3.1-*WNT5B* were synthesized by GeneCreate**,** China. These plasmids were used for the overexpression studies**.** siRNA-NC, siRNA-*WNT5B*, all-miRNA mimics, mimic NC, miRNA inhibitors, and inhibitor-NC were obtained from RiboBio, China. These reagents were used for the knockdown and modulation studies. The small molecule RNA sequences are detailed in Table S1.

To construct the *WNT5B* 3'UTR dual luciferase reporter vector, the full-length 3'UTR of *WNT5B* was amplified and inserted into the PGL3 reporter vector (Promega, USA) downstream of firefly gene. The resulting vector contained two ARE sites at *WNT5B* 3'UTR position 1608 with A or G. To mutate the ARE sites, ATTTA < ATGTA mutations were introduced using overlap extension PCR and homologous recombination. The sequences used in this process are listed in Table S2.

### Luciferase activity assay

In the experiment, cells were transfected with a total of 2 μg each of the luciferase reporter constructs using LipofectaminTM 3000 (Thermo Fisher, USA). Transfection was performed according to the manufacturer's instructions. After 36 h of transfection, the cells were harvested, and luciferase activity was analyzed using the Dual Luciferase Reporter Assay System (Promega, USA). Firefly luciferase was used as the primary reporter to monitor miRNA regulation and Renilla luciferase was used as the control reporter for normalization and screening.

### Lentivirus packaging and transduction

To package lentivirus, 1.5×10^6^ HEK293T cells were seeded in complete medium and incubated overnight at 37°C until reaching 70% confluence at 24 h. The next day, the target plasmids psPAX2 and PMD2.G (Addgene, USA) was mixed with Opti-MEM according to the corresponding ratios for transfection. The transfection complex was then added dropwise to the wells containing the HEK293T and incubated for 72 h at 37°C with CO_2_. After 72 h, the viral supernatants were collected. The harvested supernatant was centrifuged at 500✕g for 4 min to remove cell debris. The resulting supernatant was filtered using a 0.45 μm low-protein retention syringe filter (Sartorius, France), and 0.5 mL aliquots were stored at -80°C.

For lentivirus transduction, lentiviral particles containing the target gene (500 uL) were mixed with complete medium (500 uL). This mixture was added to each well of a twelve-well plate containing transduced cells. Polybrene (1 uL), a co-staining reagent, was also add to enhance transduction efficiency. The plates were incubated at 37°C with CO_2_ for 60 h.

### Real-time quantitative PCR (qRT-PCR)

Cytoplasmic, nuclear, and tissue RNA were extracted using TRIzol (Invitrogen, China) and a Cytoplasmic, Nuclear RNA Extraction Kit (Norgen Biotek, USA). cDNA reverse transcription of mRNA was performed using the HiScript III 1st Strand cDNA Synthesis kit (+gDNA wiper) (Vazyme, China) following the manufacturer's instructions. qRT-PCR was performed using Fast ChamQ Universal SYBR qPCR Master Mix (Vazyme, China) according to the manufacturer's instructions. The relative expression of each example was calculated using the 2^-ΔΔCT^ method. *NEAT1* is a known nuclear lncRNA and GAPDH is a cytoplasmic-enriched gene. The primer sequences are listed in Table S3.

### Western blot

Protein samples were extracted from treated cells or tissues using a protein lysis buffer consisting of RIPA buffer (Beyotime Biotechnology, China) and PMSF (Solarbio, China). Concentrations of the extracted proteins were determined using the BCA kit (Beyotime Biotechnology, China). To denature the protein samples, sodium dodecyl sulfate (SDS; CWBIO, China) was added and the samples were heated at 100°C for 20 min. Precast 10% and 12% sodium dodecyl sulfate-polyacrylamide gel electrophoresis (SDS-PAGE) gels (EpiZyme, China) were used with a sample volume of 10 µL. After electrophoresis, the proteins were transferred onto a 0.45 μm hybridized nitrocellulose filter membrane (NC, Merck & Co, USA) and sealed with 5% skimmed milk powder. The membrane was then incubated with the primary and secondary antibodies. Primary antibodies, including WNT5B (1:1000, Abcam, ab164311, Cambridge, UK), Cyclin A2 (1:2000, Abcam ab181591, Cambridge, UK), MYH4 (1:1000, Proteintech 20140-1-AP, China) and MYH7 (1:1000, Proteintech 22280-1-AP, China), BAX (1:1000, Abcam ab32503, Cambridge, UK), BCL2 (1:2000, Abcam ab182858, Cambridge, UK), and GAPDH (1:1000, Abcam ab9484, Cambridge, UK). Secondary antibodies were used HRP-labeled Goat Anti-Mouse IgG(H+L) (1:1000, Beyotime, A0216, China) and HRP-labeled Goat Anti-Rabbit IgG(H+L) (1:1000, Beyotime, A0208, China). Primary and secondary antibodies were diluted in 1× Tween (TBST) buffer (EpiZyme, China) as per the manufacturer's instructions. The grayscale values of the protein bands were analyzed using ImageJ software (NIH, USA).

### Cell Counting Kit-8 assay

The Cell Counting Kit-8 assay (CCK-8) was performed to measure cell proliferation in primary porcine skeletal muscle cells and C2C12 myoblasts, which were seeded in a 96-well plate for transfection. The Cell Counting Kit-8 (Beyotime Biotechnology, China) was used to detect cell growth at 0 h, 24 h, 36 h, 48 h, and 72 h post-transfection. For the assay, a mixture of CCK-8 reagent and complete culture medium was prepared in a 1:9 ratio and added to the wells. The plate was incubated at 37°C for 40 minutes. Subsequently, the absorbance at 450 nm was measured using a microplate reader, and the optical density (OD) values obtained were plotted to create a growth curve.

### 5-Ethynyl-2'-deoxyuridine (EdU) staining

Cells were cultured in 12-well plates and when they reached 50% confluence, they were transfected with plasmids, siRNA, miRNA mimics, miRNA inhibitor or corresponding controls. After 24 h of transfection, the cells were treated with 50 μM EdU (Beyotime Biotechnology, China) and incubated at 37°C for 1 or 2 h. Following this, the cells were fixed with 4% paraformaldehyde for 30 min, neutralized with a 2 mg/mL glycine solution and washed with 0.5% Triton X-100. The cells were then incubated with EdU working solution as per the manufacturer's instructions for 30 min at room temperature. Hoechst 33342 (Beyotime Biotechnology, China) was added to visualize the cell nuclei. The number of EdU-stained cells was determined using a confocal fluorescence microscope (A1HD25; Nikon, Japan). Three areas were randomly selected for the analysis.

### Cell cycle assay

After 48 h of transfection, cells were collected and fixed with 70% ethanol at 4°C for 2 h. Cells were then stained with a solution containing propidium iodide (0.05 mg/mL), RNase A (1 mg/mL), and 0.3% Triton X-100 for 30 min in the dark. To determine the percentage of cells in different cell cycle phases, DNA content (propidium iodide staining) was measured using a flow cytometer (CytoFLEX, USA). The G1, S, and G2/M phase cell populations were analyzed using ModFitLT5 software (Verity Software House, USA). A total of 10,000 cells were analyzed per sample. Each experiment was repeated three times.

### Apoptosis assay

Apoptosis was assessed using the FITC Annexin V Apoptosis Detection Kit (Yeasen Biotech, China) and analyzed by flow cytometry. Briefly, cells were harvested by digestion with EDTA-free trypsin 48 h after transfection. Double staining was performed using FITC Annexin V and propidium iodide according to the manufacturer's instructions, followed by flow cytometry (CytoFLEX, USA). Fluorescence data were analyzed using FlowJo software (Becton, Dickinson & Company, USA) for a total of 10,000 cells per sample. Each experiment was repeated three times.

### Immunofluorescence staining

Porcine primary myoblasts were induced to differentiate for four days using 5% horse serum. The cells were incubated overnight at 4°C with MYH4 (1:1000, Proteintech, 20140-1-AP, China) and MYH7 (1:1000, Proteintech, 22280-1-AP, China) primary antibodies. Fluorescent secondary antibodies (FITC-conjugated Donkey Anti-Rabbit IgG (H+L) (1:200, GB22403, Servicebio, China) and Cy3 conjugated Donkey Anti-Mouse IgG (H+L) (1:200, GB21401, Servicebio, China) were then used in combination with the primary antibodies. After DAPI staining of cell nuclei, images were acquired using a fluorescent confocal microscope (A1HD25; Nikon, Japan). For immunofluorescence of tissue sections, 10 µm sections were incubated overnight at 4°C with laminin (1:1000, Abcam, ab11575, Cambridge, UK), MYH4 (1:1000, Proteintech, 20140-1-AP, China) and MYH7 (1:1000, Proteintech, 22280-1-AP, China) primary antibodies. Fluorescent secondary antibodies (FITC-conjugated Donkey Anti-Rabbit IgG (H+L) (1:200; Servicebio, GB22403, China) and Cy3 conjugated Donkey Anti-Mouse IgG (H+L) (1:200; Servicebio, GB21401, China) were used in combination with the primary antibody. After DAPI staining of cell nuclei, images were acquired using a fluorescence confocal microscope (A1HD25; Nikon, Japan).

### Succinate Dehydrogenase (SDH) staining

Frozen sections (10 µm) were immersed in a 0.2 M sodium phosphate buffer solution (pH = 7.6) containing 0.6 mM nitro blue tetrazolium and 50 mM sodium succinate (Sigma-Aldrich, USA). The sections were then incubated at 37°C for 30 min. Images were captured using a confocal fluorescence microscope (A1HD25; Nikon, Japan).

### Statistical analysis

GraphPad Prism 9.0 software (GraphPad Software, USA) was used for statistical analysis. All experiments were repeated at least three times. All data are expressed as means ± S.E.M and analyzed with an unpaired Student's t test for statistical significance. *p* <0.05 was considered statistically significant. *p < 0.05, **p < 0.01, ***p < 0.001, p ≥ 0.05: ns (Not significant).

## Results

### *WNT5B* is a potential regulator for myogenesis

**Figure 1:**
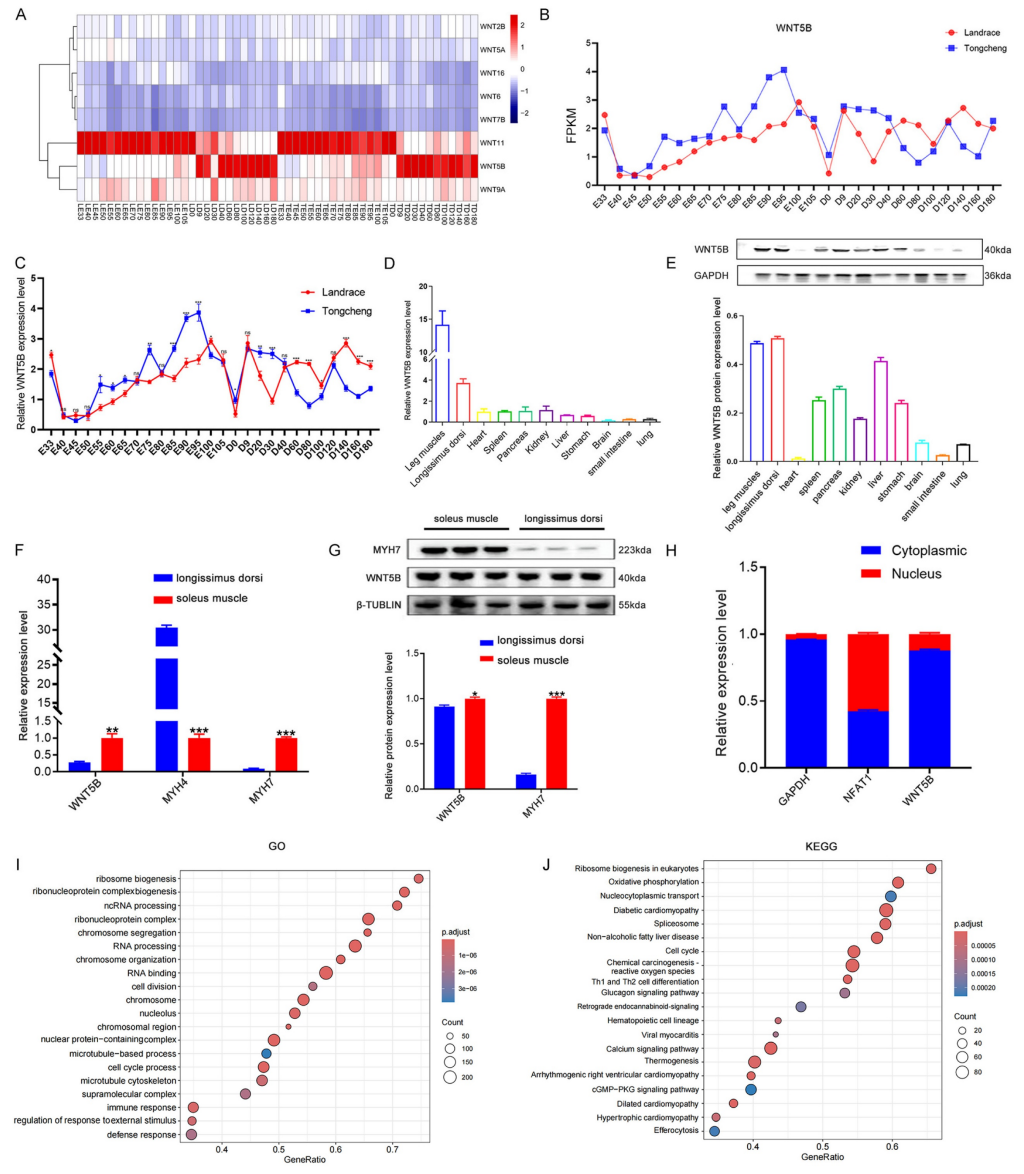
** Expression patterns of *WNT5B* gene.** (A) The heatmap of expression pattern of WNT family-related genes in developmental skeletal muscle across 27 time points in Landrace and Tongcheng pigs (LE: the embryonic stage of Landrace pig; LD: the postnatal day of Landrace pig; TE: the embryonic stage of Tongcheng pig; TD: the postnatal day of Tongcheng pig) based on the RNA-seq data. (B) RNA-seq analysis the expression level of *WNT5B* changes at 27 different developmental time points. (C) qRT-PCR result for the expression pattern of *WNT5B* in skeletal muscle from Landrace and Tongcheng pigs across 27 developmental time points. (D-E) The expression of *WNT5B* at mRNA (D) and protein (E) level in eleven different tissues of Tongcheng pigs. (F-G) The expression of *WNT5B* at mRNA (F) and protein (G) level in fast muscle fibers and slow muscle fibers. (H) qRT-PCR analysis *WNT5B* expression in the cytoplasm and nucleus of Landrace pig myoblasts. (I-J) GO (I) and KEGG (J) analysis of *WNT5B* co-expressed genes. Data are presented as mean ± SEM and analyzed for statistical differences between groups using unpaired two-tailed t-tests. *p < 0.05, **p < 0.01, ***p < 0.001, ns means no significant differences.

To investigate the regulatory role of WNT family genes in porcine myogenesis, we conducted RNA-seq analysis and observed the differential expression of *WNT5B* and *WNT11* at different developmental stages (Figure 1A). Additionally, *WNT5B* was differentially expressed at 27 time points during skeletal muscle development in Tongcheng and Landrace pigs, whereas *WNT11* was not (Figure 1B and S1A). The qRT-PCR results further confirmed that *WNT5B* was differentially expressed at different time points in Tongcheng and Landrace pigs (Figure 1C). Tissue expression analysis revealed that *WNT5B* displayed specific highly expressed in the leg muscle and longissimus dorsi (Figure 1D-E). Moreover, *WNT5B* was highly expressed in the soleus muscle (Figure 1F-G), and mainly in the myoblast cytoplasm of Tongcheng and Landrace pigs (Figure 1H and S1B). To further explore the *WNT5B* function, GO and KEGG analyses were performed, revealing that *WNT5B* and its co-expressed genes were associated with various biological processes and signaling pathways, including chromosome organization, cell division, the cGMP-PKG signaling pathway, the glucagon signaling pathway, and the cell cycle (Figure 1I-J). Furthermore, the GO terms and KEGG pathways were significantly enriched in the cell cycle, which is directly related to skeletal muscle cell proliferation, regeneration, myogenesis, and development 41-43. These results suggest that *WNT5B* is a candidate factor involved in the regulation of skeletal muscle development.

### *WNT5B* regulates cell proliferation and cell cycle

**Figure 2:**
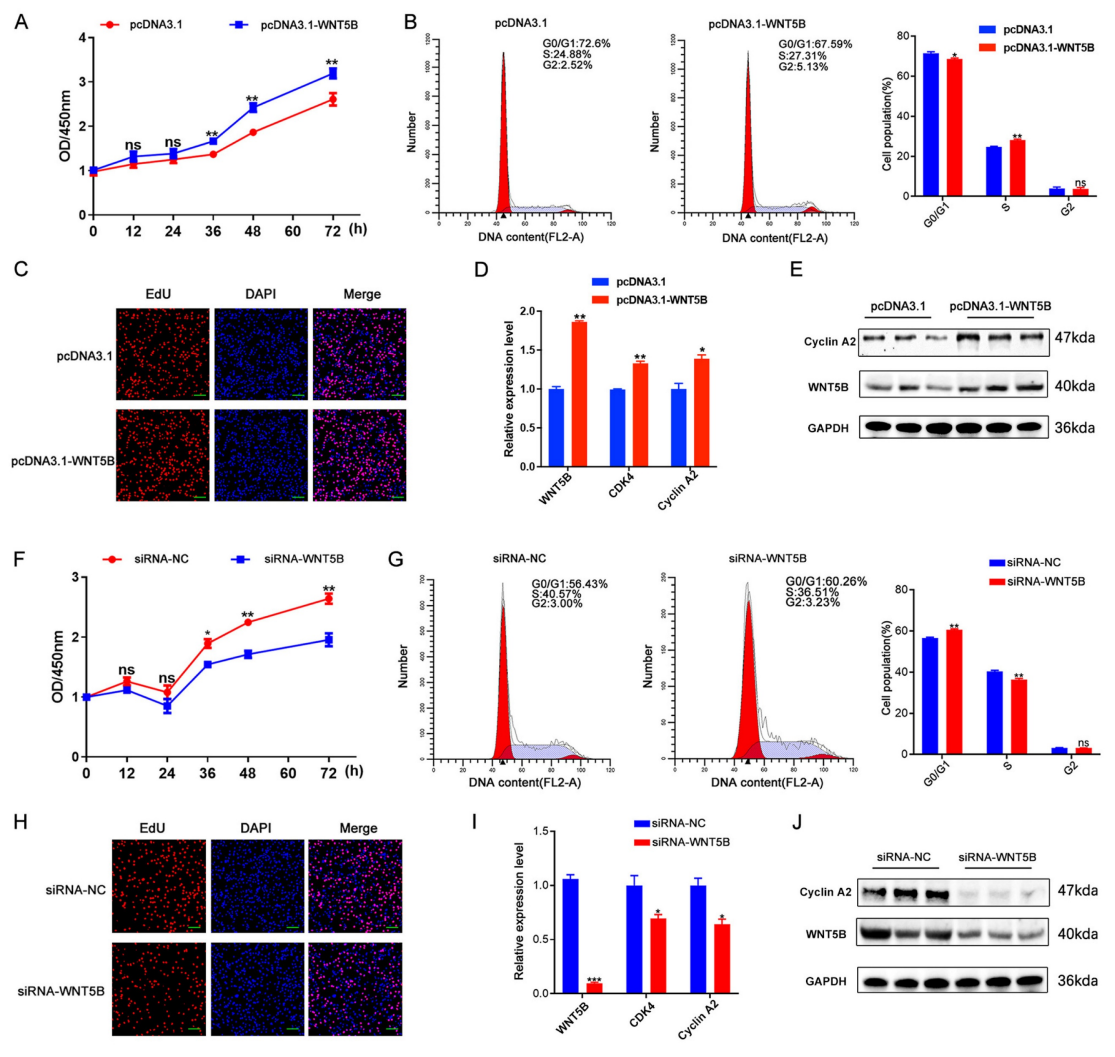
***WNT5B* promotes skeletal muscle cell proliferation and cycle in porcine.** (A-C) The results of CCK-8 (A), cell cycle (B) and cell proliferation status (C) of porcine skeletal muscle cells after transfection with pcDNA3.1 and pcDNA3.1-*WNT5B* vectors. Scale bar, 50 μm. (D-E) mRNA (D) and protein (E) expression levels of proliferation marker genes in porcine skeletal muscle cells after *WNT5B* overexpression. (F-H) The results of CCK-8 (F), cell cycle (G) and cell proliferation status (H) of porcine skeletal muscle cells after transfection with siRNA-NC and siRNA-*WNT5B*. Scale bar, 50 μm. (I-J) mRNA (I) and protein (J) expression levels of proliferation marker genes in porcine skeletal muscle cells after *WNT5B* knockdown. Data are presented as mean ± SEM and analyzed for statistical differences between groups using unpaired two-tailed t-tests. *p < 0.05, **p < 0.01, ***p < 0.001, ns means no significant differences.

The effects of *WNT5B* on the proliferation and cell cycle of porcine myoblasts were subsequently examined. To elucidate its functional role, siRNA and overexpression vectors targeting *WNT5B* were constructed and transfected into porcine myoblasts. The results showed that *WNT5B* overexpression significantly decreased the percentage of cells in the G0/G1 phase and increased the percentage of cells in the S phase, as well as the number of proliferating and EdU-positive cells (Figure 2A-C). Moreover, *WNT5B* overexpression upregulated cell proliferation and the expression of cell cycle markers (*CDK4* and Cyclin A2) (Figure 2D-E). Conversely, knockdown of *WNT5B* resulted in the opposite effects compared to its overexpression (Figure 2F-J). Sequence conservation analysis revealed that *WNT5B* is highly conserved in pigs and mice (Figure S2A). Consequently, the effects on cell proliferation and cell cycle were examined by transfecting porcine *WNT5B* overexpression vectors and siRNA into C2C12 myoblasts. The results showed that the effects of knockdown and overexpression of *WNT5B* in C2C12 myoblasts on cell proliferation and the cell cycle were consistent with those observed in porcine myoblasts. Overexpression of *WNT5B* promoted cell proliferation and cell cycle progression, while knockdown of *WNT5B* inhibited the proliferation and cell cycle progression of C2C12 myoblasts (Figure S3). These results suggest that *WNT5B* regulates myogenesis and demonstrates functional conservation across species.

### *WNT5B* regulates cell apoptosis in myoblasts

**Figure 3:**
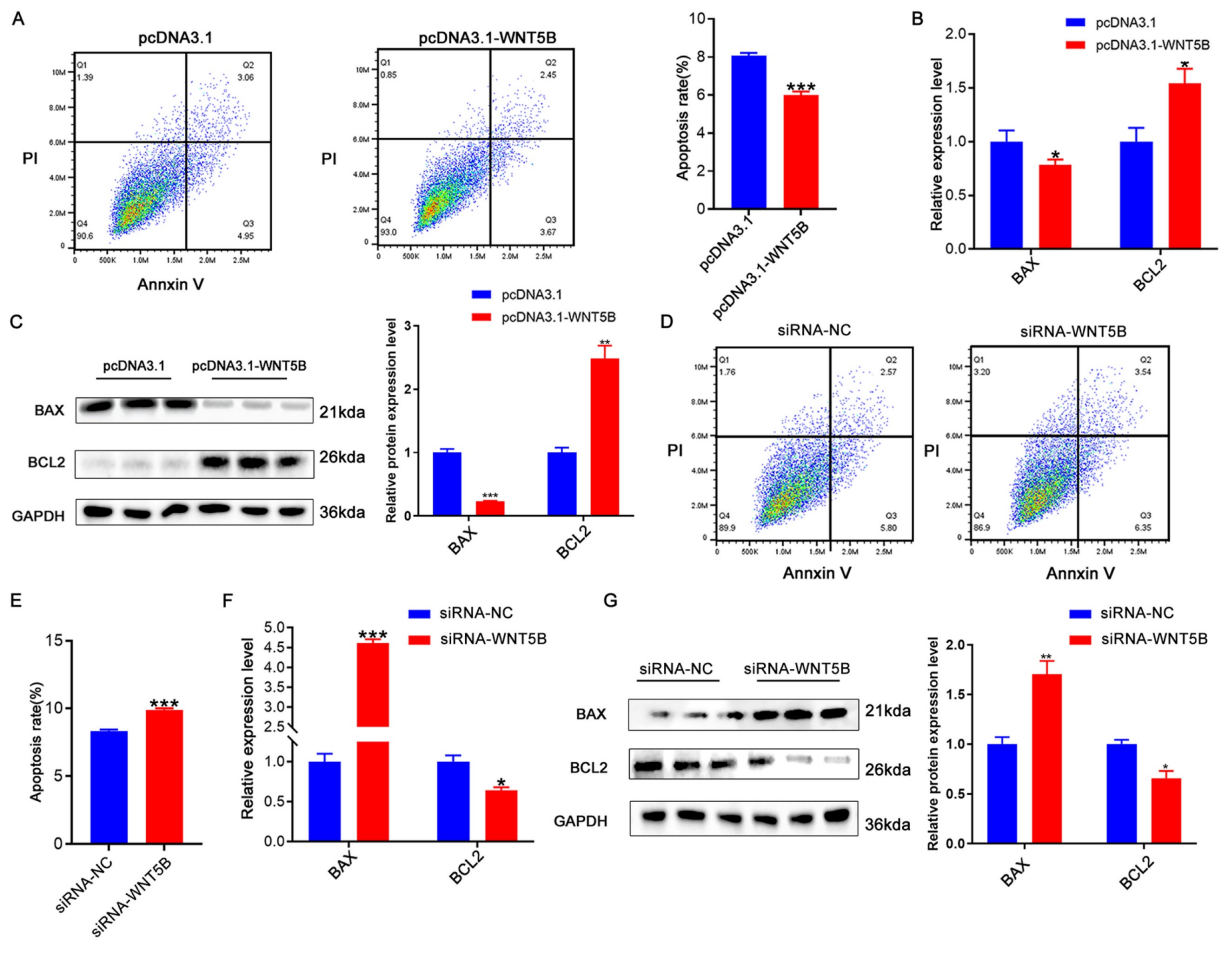
** The effects of *WNT5B* on cell apoptosis.** (A) The results of cell apoptosis of porcine skeletal muscle cells after *WNT5B* overexpression. (B-C) mRNA (B) and protein (C) expression levels of cell apoptosis markers genes in porcine skeletal muscle cells after *WNT5B* overexpression. (D-E) The results of cell apoptosis of porcine skeletal muscle cells after *WNT5B* knockdown. (F-G) mRNA (F) and protein (G) expression levels of cell apoptosis marker genes in porcine skeletal muscle cells after *WNT5B* knockdown. Data are presented as mean ± SEM and analyzed for statistical differences between groups using unpaired two-tailed t-tests. *p < 0.05, **p < 0.01, ***p < 0.001, ns means no significant differences.

To investigate the role of *WNT5B* in myogenesis, we examined its effect on apoptosis. Overexpression of *WNT5B* led to a reduction in apoptosis rates in porcine and C2C12 myoblasts (Figure 3A and S4A). Moreover, at both the mRNA and protein levels, *WNT5B* overexpression resulted in decreased *BAX* expression and increased *BCL2* expression (Figure 3B-C and S4B-C). Conversely, *WNT5B* knockdown promoted apoptosis in porcine and C2C12 myoblasts (Figure 3D-E and S4D-E). *BAX* expression was upregulated, while *BCL2* expression was downregulated (Figure 3F-G and S4F-G). Collectively, these results suggest that *WNT5B* promotes cell proliferation and cell cycle progression and suppresses myoblast apoptosis.

### *WNT5B* regulates fiber type conversion of porcine skeletal muscle

**Figure 4:**
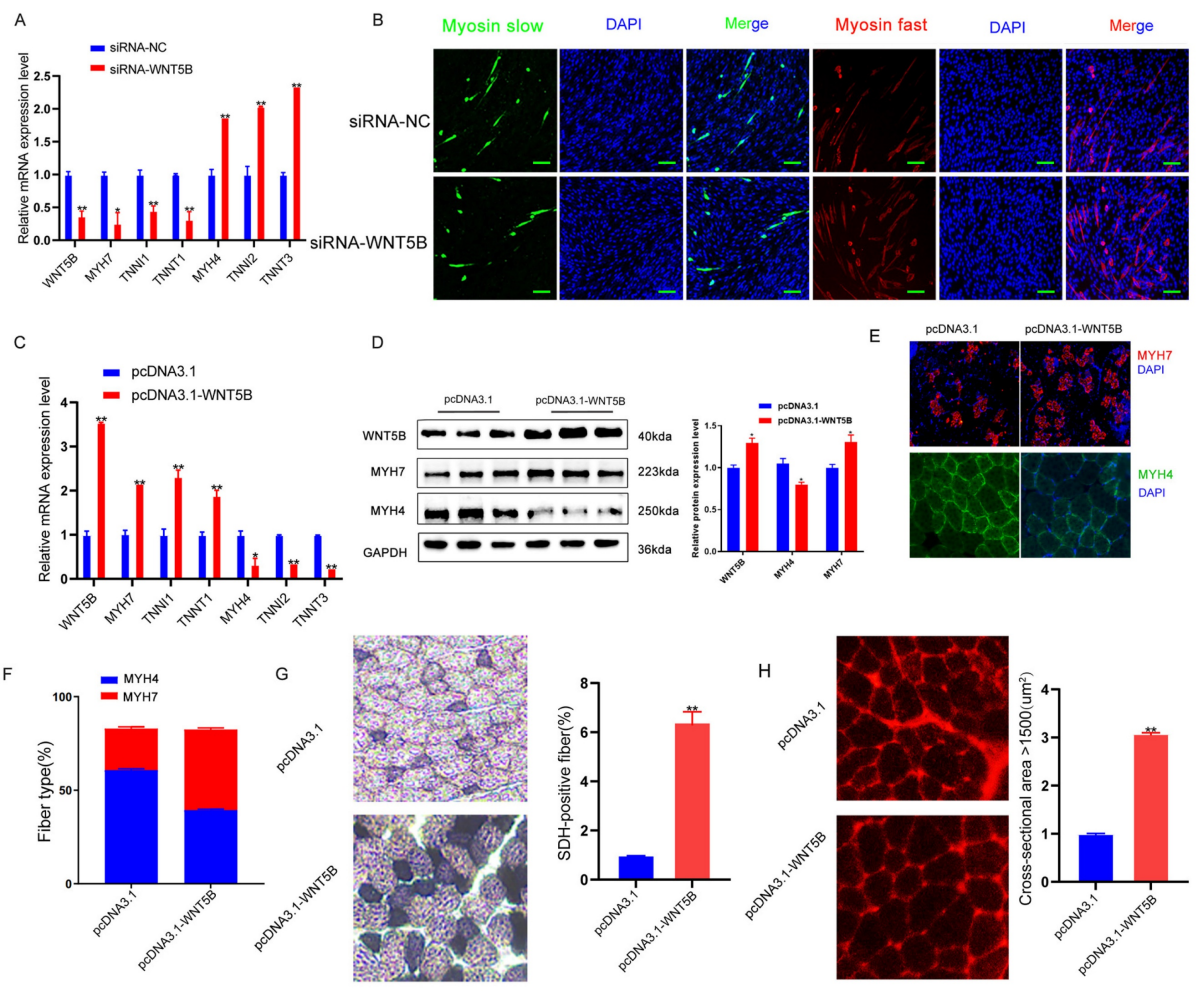
** The effects of *WNT5B* on the muscle fiber type conversion.** (A) The mRNA expression level of muscle fiber type transformation markers in porcine primary myoblast after *WNT5B* knockdown. (B) Immunofluorescence staining results of slow and fast muscle fiber transformation in porcine primary myoblasts after *WNT5B* knockdown. Scale bar, 50 μm. (C-D) The results of *WNT5B* overexpression of mRNA (C) and protein (D) expression levels of muscle fiber type transformation markers *in vivo*. (E-F) Immunofluorescence (E) results of the regeneration of slow and fast fibers after overexpression of *WNT5B in vivo*. Scale bar, 20 μm. The quantity (F) results of slow- and fast-fibers regeneration. (G) Succinate dehydrogenase (SDH) staining results of the slow muscle fiber formation after *WNT5B* overexpression *in vivo*. (H) Laminin staining results of muscle fiber thickness after *WNT5B* overexpression* in vivo*. Data are presented as mean ± SEM and analyzed for statistical differences between groups using unpaired two-tailed t-tests. *p < 0.05, **p < 0.01, ***p < 0.001, ns means no significant differences.

The results presented in Figure 1F-G suggest that *WNT5B* may play a role in regulating the conversion of myofiber types. The effects of *WNT5B* on pig myofiber myogenesis were examined. In *vitro*, knockdown of *WNT5B* down-regulated the expression of slow muscle-specific genes (*MYH7*, *TNNI1*, and *TNNT1*) and upregulated the expression of fast muscle-specific genes (*MYH4*, *TNNI2*, and *TNNT3*) (Figure 4A). Immunofluorescence experiments confirmed that *WNT5B* knockdown inhibited the generation of slow muscle fiber (Figure 4B). Furthermore, the *WNT5B* overexpression vector was packaged using a lentivirus. Its impact on the myofiber area and myofiber type conversion in porcine skeletal muscle was examined 28 days post-injection into quadriceps. In *vivo*, slow muscle-specific genes (*MYH7*, *TNNI1*, and *TNNT1*) were upregulated and fast muscle-specific genes (*MYH4*, *TNNI2*, and *TNNT3*) downregulated after WNT5B overexpression (Figure 4C). Western blot and immunofluorescence assays further revealed that *WNT5B* overexpression resulted in increased* MYH7* expression, decreased *MYH4* expression, and an increase in the number of slow muscle fibers* in vivo* (Figure 4D-F). Additionally, SDH and Laminin staining demonstrated that *WNT5B* overexpression promoted the formation of slow fibers and increased the muscle fiber area *in vivo* (Figure 4G-H). These results indicate that *WNT5B* plays a significant role in fiber type conversion.

### The SNP c.1608 A > G in the 3'UTR affects *WNT5B* mRNA stability

**Figure 5:**
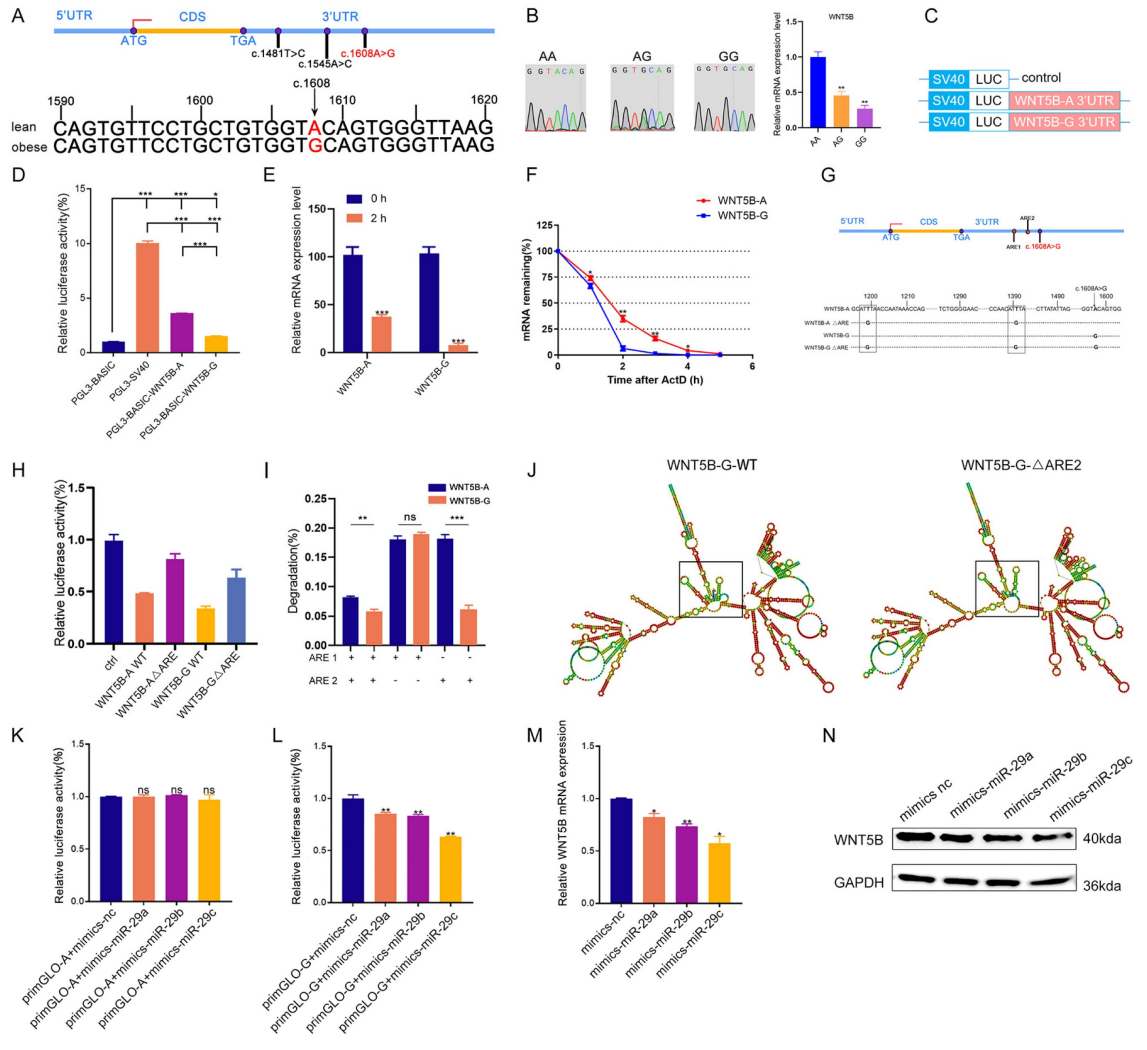
** Effects of SNPs on *WNT5B* mRNA stability.** (A) The sequencing results indicate the 3'UTR of the *WNT5B* gene in obese-type and lean-type pigs. A SNP site, named c.1608 A > G. (B) The mRNA expression of *WNT5B* in AA, AG, GG genotype pigs. (C) Schematic of a dual-luciferase reporter vector construction containing *WNT5B*-A-3'UTR or *WNT5B*-G-3'UTR vectors. (D) Dual-luciferase activity was analyzed after transfection with *WNT5B*-A-3'UTR or *WNT5B*-G-3'UTR vector in HEK-293T cells. (E) The luciferase mRNA expression level at 2 hours after transfection of *WNT5B*-A and *WNT5B*-G. (F) The expression levels of *WNT5B*-A and *WNT5B*-G at various time points following treatment with actinomycin D. (G) Positional pattern of the ARE sites on the *WNT5B* 3'UTR. (H-I) The results of the dual-luciferase activity assay after mutation ARE sites in *WNT5B*-A-3'UTR or *WNT5B*-G-3'UTR vectors. (J) The effects of WT and ARE2 (right) site mutations on *WNT5B*-G mRNA secondary structure. (K-L) Dual-luciferase were used to analyze the effect of miR-29a/b/c on the SNP (c.1608 A > G) in *WNT5B*. (M-N) Expression levels of *WNT5B* mRNA (M) and protein (N) after miR-29a/b/c overexpression in porcine myoblasts. Data are presented as mean ± SEM and analyzed for statistical differences between groups using unpaired two-tailed t-tests. *p < 0.05, **p < 0.01, ***p < 0.001, ns means no significant differences.

Given WNT5B gene varied in the transcriptomic data from 27 different developmental time points in the skeletal muscle of obese and lean pig breeds, we aimed to investigate the reasons behind the differences. Gene expression is influenced by various regulatory factors, and the 3'UTR sequence contains key elements that regulate the spatial and temporal expression of gene mRNAs, thereby determining cellular fates 44, 45. Previous studies have demonstrated the association between genetic variations in the 3'UTR and livestock and poultry production performance 46, 47. This study investigated whether there was SNP site in the 3'UTR of the *WNT5B* gene that could regulate its expression. The 3'UTR of the *WNT5B* gene was amplified in different breeds of obese-type (Tongcheng, Bama, Luchuan) and lean-type (Landrace) porcine, and a SNP site, named c.1608 A > G, was identified at position 1608 of the *WNT5B* 3'UTR. *WNT5B* was most highly expressed in the AA genotype, followed by the AG genotype, and least highly expressed in the GG genotype (Figure 5A-B). To further understand the effects of the c.1608 A > G SNP on the post-transcriptional regulation and mRNA stability of *WNT5B*, a dual-luciferase vector was constructed containing the full-length sequence of *WNT5B* 3'UTR with either A (*WNT5B*-A) or G (*WNT5B*-G) at the SNP site (Figure 5C). The dual-luciferase activity assay results revealed that the luciferase activity of the *WNT5B*-G 3'UTR was 30-40% lower than that of the *WNT5B*-A 3'UTR (Figure 5D). Furthermore, analysis of luciferase mRNA levels showed that luciferase mRNA containing the *WNT5B*-G 3'UTR decayed twice as fast as mRNA containing the *WNT5B*-A 3'UTR, indicating that a single-nucleotide change at the 3'UTR of *WNT5B* affects the overall stability of mRNA transcripts (Figure 5E-F).

The 3'UTR of *WNT5B* contains two adenosine-uridine repeats (AREs) in the *cis*-regulatory region, which are known to be involved in mRNA stability by binding to degradative RNA-binding proteins (Figure 5G). To investigate the role of AREs in the differential regulation of *WNT5B*-A and *WNT5B*-G, dual-luciferase expression vectors were constructed: one with the wild-type sequence and the other with a mutation that altered the original AUUUA sequence to AUGUA. The dual-luciferase activity assays showed that the *WNT5B*-A ΔARE and *WNT5B*-G ΔARE 3'UTRs exhibited similar rescue efficiency compared to the *WNT5B*-A and *WNT5B*-G 3'UTRs (Figure 5H). Furthermore, the differential regulation of *WNT5B*-A versus *WNT5B*-G was maintained in the presence of ARE2 alone, independent of ARE1 (Figure 5I-J and S5).

### miR-29a/b/c regulates myogenesis

Recent studies have shown that miRNAs collaborate with ARE-binding proteins to destabilize cytokines 48-50, miRNA play a role in silencing target genes by recognizing the 3'UTR region of target mRNAs and forming a silencing complex 51*.* This study further analyzed whether the *WNT5B* 3'UTR SNP affects miRNA binding. Interestingly, this SNP was located in the MRE (microRNA Response Elements) region of miR-29a/b/c (Figure S6A). Additionally, miR-29a/b/c sequences were conserved among various species (Figure S2B). Dual-luciferase activity assays confirmed that miR-29a/b/c bound to the G site but not to the A site (Figure 5K-L). Following overexpression of miR-29a/b/c into WNT5B-G porcine myoblasts, both mRNA and protein levels of *WNT5B* were downregulated, indicating that miR-29a/b/c can indeed bind to the *WNT5B* 3'UTR (Figure 5M-N).

**Figure 6:**
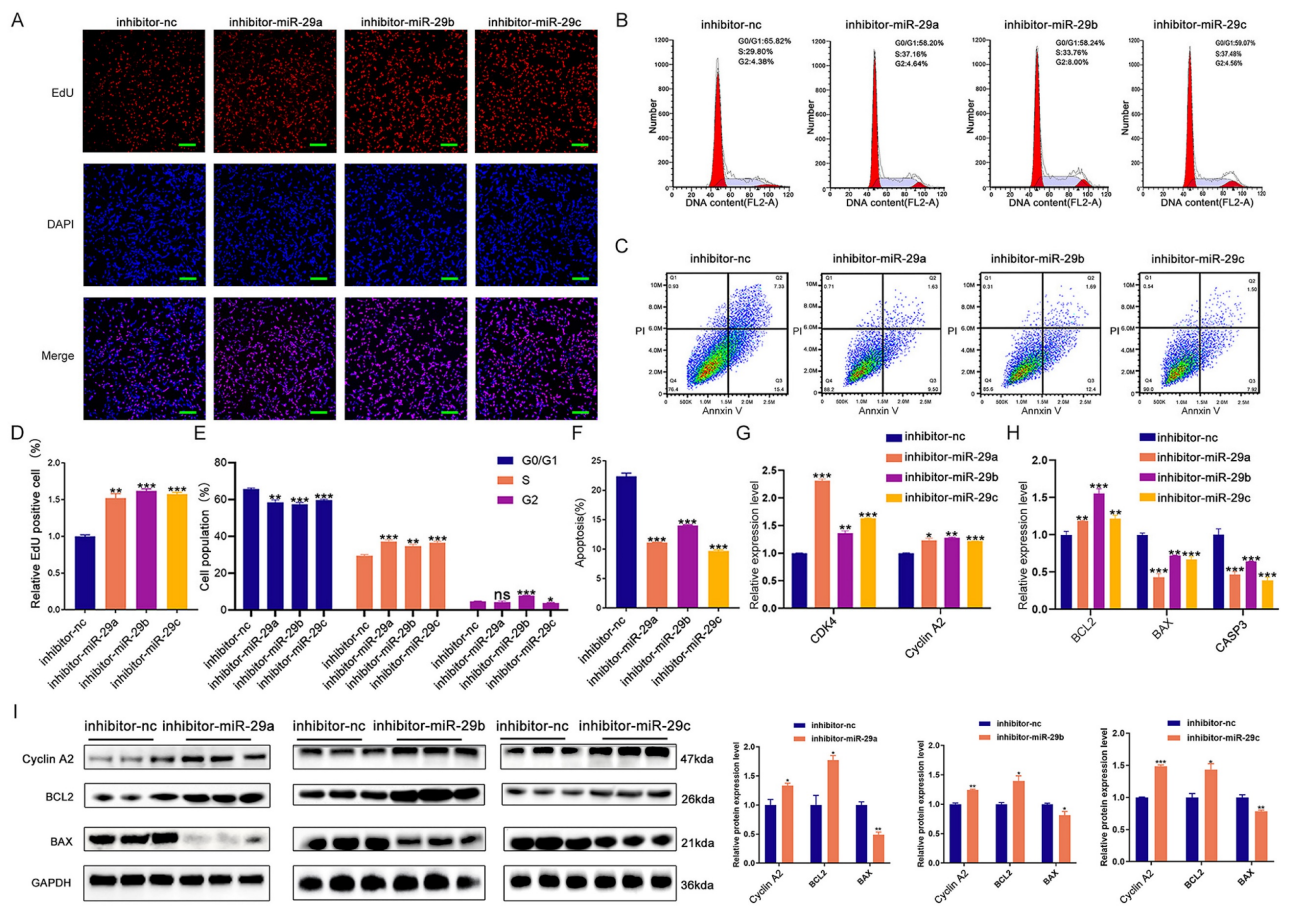
** The effects of miR-29a/b/c knockdown on proliferation and apoptosis in porcine skeletal muscle cells.** (A-C) The results of cell proliferation (A), cell cycle (B), and cell apoptosis (C) after transfection with miR-29a/b/c inhibitor in porcine skeletal muscle cells. Scale bars, 50 μm. (D-F) Quantitative results of cell proliferation (D), cell cycle (E), and cell apoptosis (F). (G-H) The mRNA expression of cell cycle (G) and cell apoptosis (H) markers expression after miR-29a/b/c knockdown in porcine skeletal muscle cells. (I) The protein expression of cell cycle and cell apoptosis markers after miR-29a/b/c knockdown in porcine skeletal muscle cells. Data are presented as mean ± SEM and analyzed for statistical differences between groups using unpaired two-tailed t-tests. *p < 0.05, **p < 0.01, ***p < 0.001, ns means no significant differences.

We subsequently investigated the effects of miR-29a/b/c on cell proliferation, cell cycle, and apoptosis. EdU staining, cell cycle analysis, and apoptosis assays demonstrated that knockdown of miR-29a/b/c enhanced cell proliferation and cell cycle progression, while suppressing apoptosis in porcine skeletal muscle cells (Figure 6A-F and S7A-B). Additionally, inhibition of miR-29a/b/c led to increased mRNA expression of *CDK4*, Cyclin A2, and *BCL2,* and decreased expression of *BAX* and *CASP3* (Figure 6G-H). Western blot analysis further revealed that knockdown of miR-29a/b/c resulted in elevated expression of Cyclin A2 and *BCL2* proteins, as well as decreased expression of *BAX* protein (Figure 6I). Overexpression of miR-29a/b/c had effects on cell proliferation, cell cycle, and apoptosis that were opposite to those observed with knockdown (Figure S8A-I). Furthermore, the binding site for miR-29a/b/c was conserved between pig and mouse in *WNT5B* 3'UTR and the function of miR-29a/b/c in C2C12 myoblasts proliferation, cell cycle and apoptosis were consistent with those observed in porcine myoblast (Figure S6B, S7C-D and S9A-L).

### miR-29a/b/c regulates myogenesis by targeting *WNT5B*-G 3'UTR

**Figure 7:**
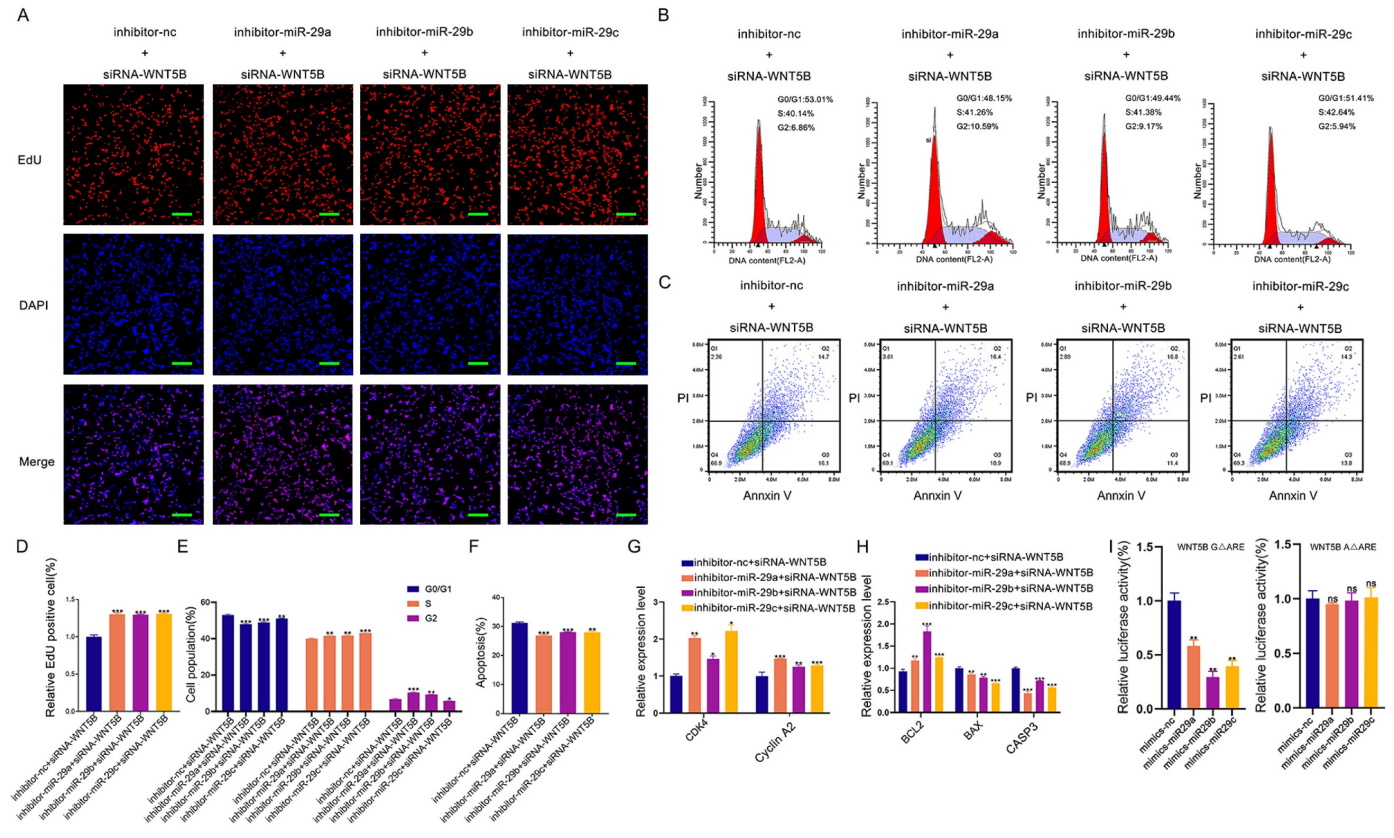
** miR-29a/b/c regulates cell proliferation and apoptosis through binding to *WNT5B*.** (A-C) The results of cell proliferation (A), cell cycle (B), and cell apoptosis (C) after miR-29a/b/c and *WNT5B* knockdown in porcine skeletal muscle cells. Scale bar, 50 μm. (D-F) Quantitative results of cell proliferation (D), cell cycle (E), and cell apoptosis (F). (G-H) The expression of cell cycle (G) and cell apoptosis markers (H) in mRNA level after co-transfection with miR-29a/b/c inhibitor and *WNT5B* siRNA in porcine skeletal muscle cells. (I) The effects of co-transfection with miR-29a/b/c mimics and mutation of ARE1 and ARE2 sites on *WNT5B*-A-3'UTR or *WNT5B*-G-3'UTR vectors. Data are presented as mean ± SEM and analyzed for statistical differences between groups using unpaired two-tailed t-tests. *p < 0.05, **p < 0.01, ***p < 0.001, ns means no significant differences.

Next, we tested whether miR-29a/b/c could regulate cell proliferation, cell cycle, and apoptosis through *WNT5B* by co-transfection experiments. Results from EdU staining, cell cycle analysis, and apoptosis assays demonstrated that miR-29a/b/c knockdown significantly impacted these cellular processes, but these effects were rescued by *WNT5B* siRNA (Figure 7A-F). Additionally, the effect of miR-29a/b/c inhibitor on the mRNA expression of *CDK4*, Cyclin A2, *BAX*, *CASP3*, and *BCL2* was reversed by *WNT5B* knockdown (Figure 7G-H). This rescue was also observed in C2C12 myoblasts (Figure S10A-H).

We further investigated whether ARE-binding sites are involved in miRNA-induced silencing complex (miRISC) recruitment. miR-29a/b/c mimics were co-transfected with *WNT5B*-A 3'UTR and *WNT5B*-G 3'UTR along with the *WNT5B* ΔARE luciferase reporter vector. Similar to the effects observed with the *WNT5B*-A and *WNT5B*-G 3'UTR, miR-29a/b/c inhibited the *WNT5B*-G ΔARE 3'UTR but had no significant effect on the *WNT5B*-A ΔARE 3'UTR (Figure 7I). Thus, miRISC recruitment to the *WNT5B*-G 3'UTR does not require an ARE-binding protein. Overall, these results indicate that miR-29a/b/c can directly target and mediate the degradation of the *WNT5B*-G 3'UTR. The A-to-G position SNP have a protective effect against inhibition by miR-29a/b/c.

## Discussion

*WNT5B* plays crucial roles in various biological processes, including osteogenic differentiation, cartilage formation, adipose differentiation, etc. 16, 52. However, its role in myogenesis has not yet been fully elucidated. A previous study indicated differential expression of *WNT5B* during myogenesis induction in the P19 cell line 53. In this study, differential expression of *WNT5B* was observed in obese and lean pigs during skeletal muscle development. Its expression was higher in the soleus muscle. These findings suggest that *WNT5B* plays an important role in skeletal muscle development.

The study demonstrates that *WNT5B* promotes the proliferation and cell cycle of porcine skeletal muscle cells. This is consistent with previous reports showing that *WNT5B* promotes cell proliferation and cell cycle progression in cancer and LAD cells 21, 36. Additionally, overexpression of *WNT5B* inhibited *CASP3/7* activity and apoptosis in HEK293T cells 54. Our study further demonstrated that *WNT5B* inhibits apoptosis in porcine skeletal muscle cells. Moreover, the effects of *WNT5B* on cell proliferation, cell cycle, and apoptosis are functionally conserved in pigs and mice. Importantly, *WNT5B* expression was higher in slow muscle fibers than in fast muscle fibers, and promoted the transformation of fast to slow fibers. Different muscle fiber types are associated with muscle thickness and meat quality 55-58. Therefore, the results suggest that *WNT5B* regulates myogenesis and plays an important role in meat quality traits.

To investigate the regulatory mechanism of *WNT5B* on myogenesis, we analyzed the SNP c.1608 A > G located at the 3'UTR of *WNT5B* in porcine. We found that the G allele at the c.1608 A > G SNP site led to a decrease in *WNT5B* mRNA stability. Additionally, the SNP reduced the miR-29a/b/c miRNA response element motif. It was discovered that miR-29a/b/c inhibited the activity of the *WNT5B*-G 3'UTR luciferase reporter vector, but had no effect on the activity of the *WNT5B*-A 3'UTR luciferase reporter vector. Moreover, the SNP attenuated the inhibitory effect of miR-29a/b/c on *WNT5B* 3'UTR activity. These findings are consistent with previous reports indicating that SNPs can alter miRNA interactions with the target 3'UTRs, resulting in reduced the degradation of the target gene 59. Previous studies have shown that SNPs located within the miRNA-binding motifs disrupt their interactions, thereby altering the expression of target genes and influencing cellular processes 60, 61. In our study, miR-29a/b/c regulated the proliferation and apoptosis of porcine and mouse skeletal muscle cells by targeting *WNT5B*.

Mutations in RNA regulatory elements have been shown to impact gene expression and function by affecting interactions between RNA-binding proteins, miRNAs, and gene 3'UTRs 61-63. For example, in the *BMP2* 3'UTR, the SNP rs15705 has been identified as a variant associated with osteoporosis 64. Furthermore, the SNP located in the ARE binding domain of *BMP2* has been shown to affect its 3'UTR activity and post-transcriptional regulation 65-67. This study examined the 3'UTR of *WNT5B* gene and classified it as an ARE-containing mRNA due to its possession of two copies of the pentameric AUUUA motif. Further experiments revealed that the inhibitory effect of miR-29a/b/c on the* WNT5B* 3'UTR was abolished upon mutation of the two ARE sites. Notably, a mutation in the second ARE site near the c.1608 A > G SNP was observed to directly rescue the luciferase activity of *WNT5B*. This outcome may be due to the alteration of ARE sites, which can influence the secondary structure of *WNT5B* mRNA. However, further studies are required to confirm these regulatory mechanisms. miRNAs cooperate with ARE-binding proteins to destabilize mRNAs that encode cytokines. AREs serve as a signal for miRNA-activated translation and under miRNA guidance, miRISC complex members such as AGO and FMRP are recruited to ARE to activate translation and regulate gene expression 68.

## Conclusions

**Figure 8:**
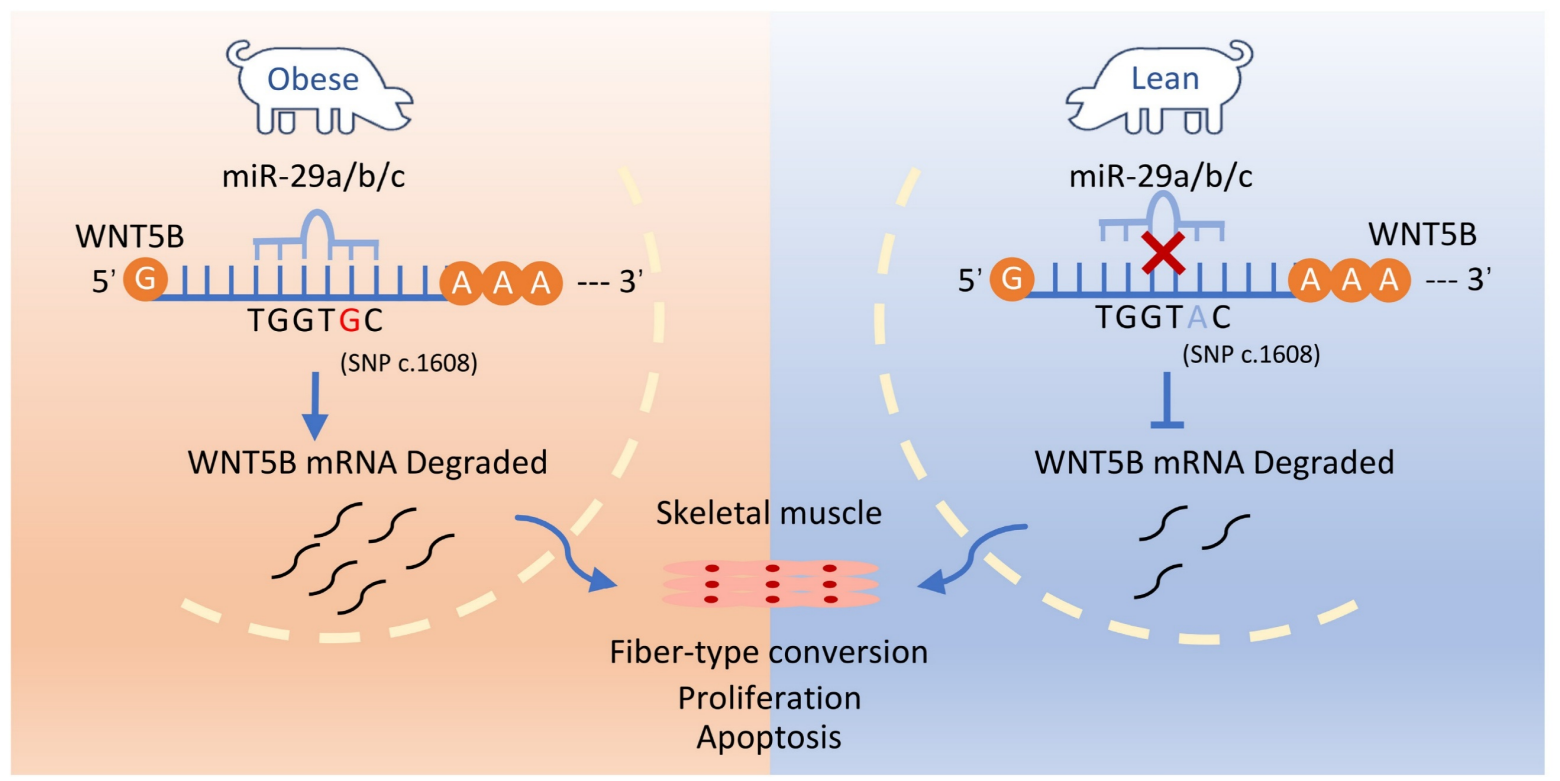
c.1608 A > G SNP protects the *WNT5B* gene 3'UTR from degradation by the miR-29 family of genes, affecting proliferation, cell cycle and apoptosis in skeletal muscle cells.

This study identified and confirmed a functional SNP site (c.1608 A > G) in the *WNT5B* 3'UTR, which influences *WNT5B* mRNA stability through the ARE binding site. This functional SNP is located within the miR-29a/b/c miRNA response element motif. The c.1608 A > G SNP protects the *WNT5B* gene 3'UTR from degradation by miR-29 family genes, leading to an increase in *WNT5B* expression. This upregulation subsequently regulates the proliferation, cell cycle, and apoptosis of skeletal muscle cells. Our results suggest that *WNT5B* has significant and promising effects on porcine skeletal muscle production traits (Figure 8).

## Supplementary Material

Supplementary figures and tables.

## Abbreviations

ARE: Adenosine-uridine Repeats Element
MRE: MicroRNA Response Elements
ATCC: American Type Culture Collection
BSA: Bovine Serum Albumin
circRNA: Circular no-coding RNA
DAPI: 4',6-Diamidino-2-phenylindole dihydrochloride
DEPC: Diethyl Pyrocarbonate
DMEM: Dulbecco's Modified Eagle Medium
DPBS: Dulbecco's Phosphate-Buffered Saline
EDTA: Ethylene Diamine Tetraacetic Acid
EdU: 5-Ethynyl-2'-deoxyuridine
FBS: Fetal Bovine Serum
GO: Gene Ontology
GSEA: Gene Set Enrichment Analysis
KEGG: Kyoto Encyclopedia of Genes and Genomes
lncRNA: Long no-coding RNA
miRISC: miRNA-induced silencing complex
miRNA: MicroRNA
MYH: Myosin Heavy Chains
PBS: Phosphate Buffered Saline
PI: Propidium Iodide
PS: Penicillin-Streptomycin
PMSF: Phenylmethanesulfonylfluoride
qRT-PCR: quantitative real-time PCR
SDS: Sodium dodecyl sulfate
siRNA: Small interfering RNA
SNP: Single nucleotide polymorphism
TBST: TBS with Tween-20
UTR: Untranslated region
WNT: Wingless-integrated
WNT5B: WNT Family Member 5B
