# Supplementary material for: WNT5B regulates myogenesis and fiber type conversion by affecting mRNA stability
Source: Int J Biol Sci. 2025 Jun 9;21(9):3934–48. doi: 10.7150/ijbs.102309 (PMC12223781; doi:10.7150/ijbs.102309)
Supplement: Supplementary file 1 — Supplementary figures and tables. [file ijbsv21p3934s1.pdf]

WNT5B regulates myogenesis and fiber type conversion by affecting mRNA stability

Danyang Fan[1,2,3#], Yilong Yao[1,2,3#], Chao Yan[1,2,3#], Fanqinyu Li[2,3], Yalan Yang[1,2,3], Bingkun Xie[4], Zhonglin Tang[1,2,3,4*]

1 Shenzhen Branch, Guangdong Laboratory for Lingnan Modern Agriculture, Agricultural Genomics Institute at Shenzhen, Chinese Academy of Agricultural Sciences, Shenzhen 518124, China

2 Kunpeng Institute of Modern Agriculture at Foshan, Agricultural Genomics Institute, Chinese Academy of Agricultural Sciences, Foshan 528226, China;

3 Key Laboratory of Livestock and Poultry Multi-Omics of MARA, Agricultural Genomics Institute at Shenzhen, Chinese Academy of Agricultural Sciences, Shenzhen 518124, China;

4 Guangxi Key Laboratory of Livestock Genetic Improvement, Guangxi Institute of Animal Sciences, Nanning, 530001, China.

[#]These authors equally contribute to this work.

*To whom correspondence should be addressed: tangzhonglin@caas.cn.

Corresponding Address: Agricultural Genomics Institute at Shenzhen, Chinese Academy of Agricultural Sciences, Pengfei Road 7, Da Peng New District, Shenzhen, Guangdong Province, China.

Supplementary Figures

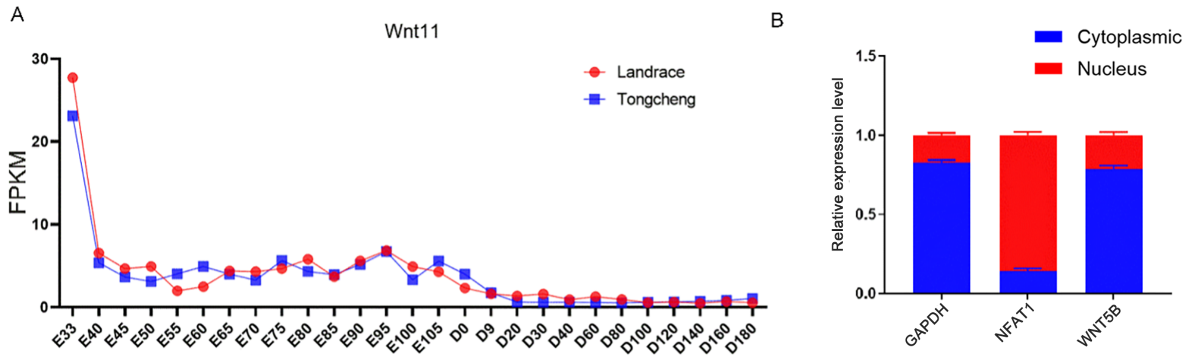

Figure S1. RNA-seq data from skeletal muscle samples of Landrace and Tongcheng pigs during both prenatal and postnatal stages

(A) RNA-seq analysis of the expression level of *WNT11* changes at 27 different developmental time points.

(B) qRT-PCR analysis of *WNT5B* expression in the cytoplasm and nucleus of Tongcheng pig myoblasts.

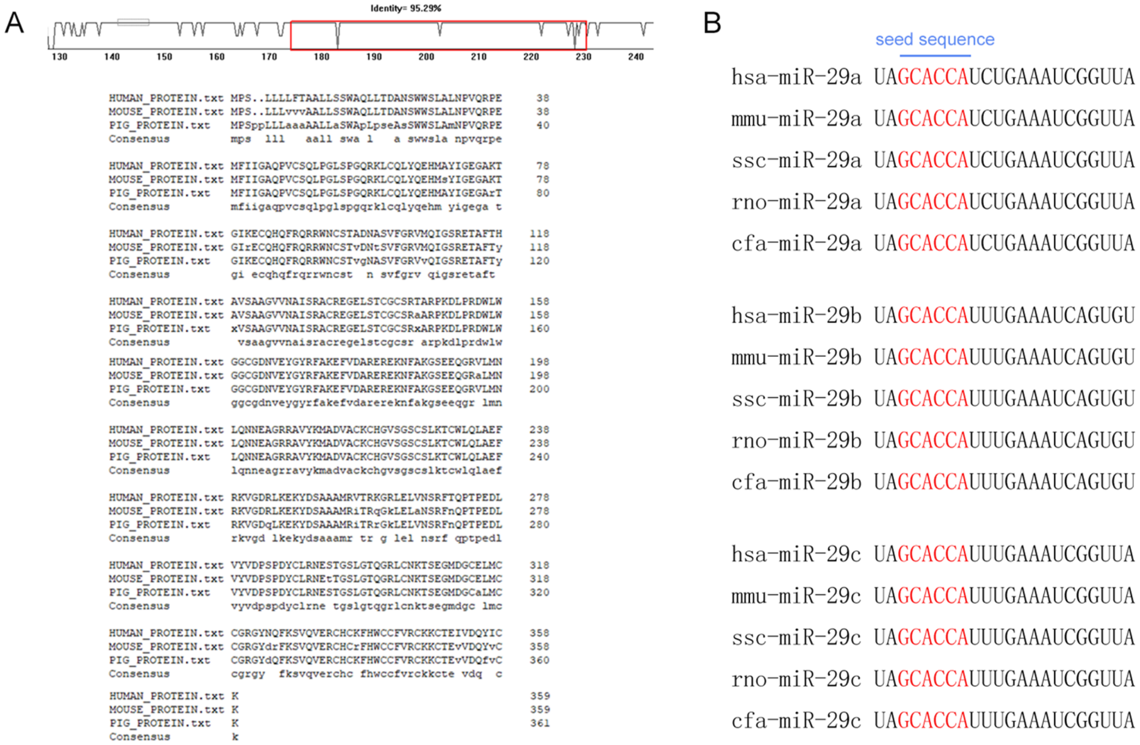

Figure S2. Analysis of *WNT5B* 3’UTR and miR-29a/b/c sequences in different species.

(A) Conservation analysis of the *WNT5B* across different species.

(B) Conservation analysis of the miR-29a/b/c across different species.

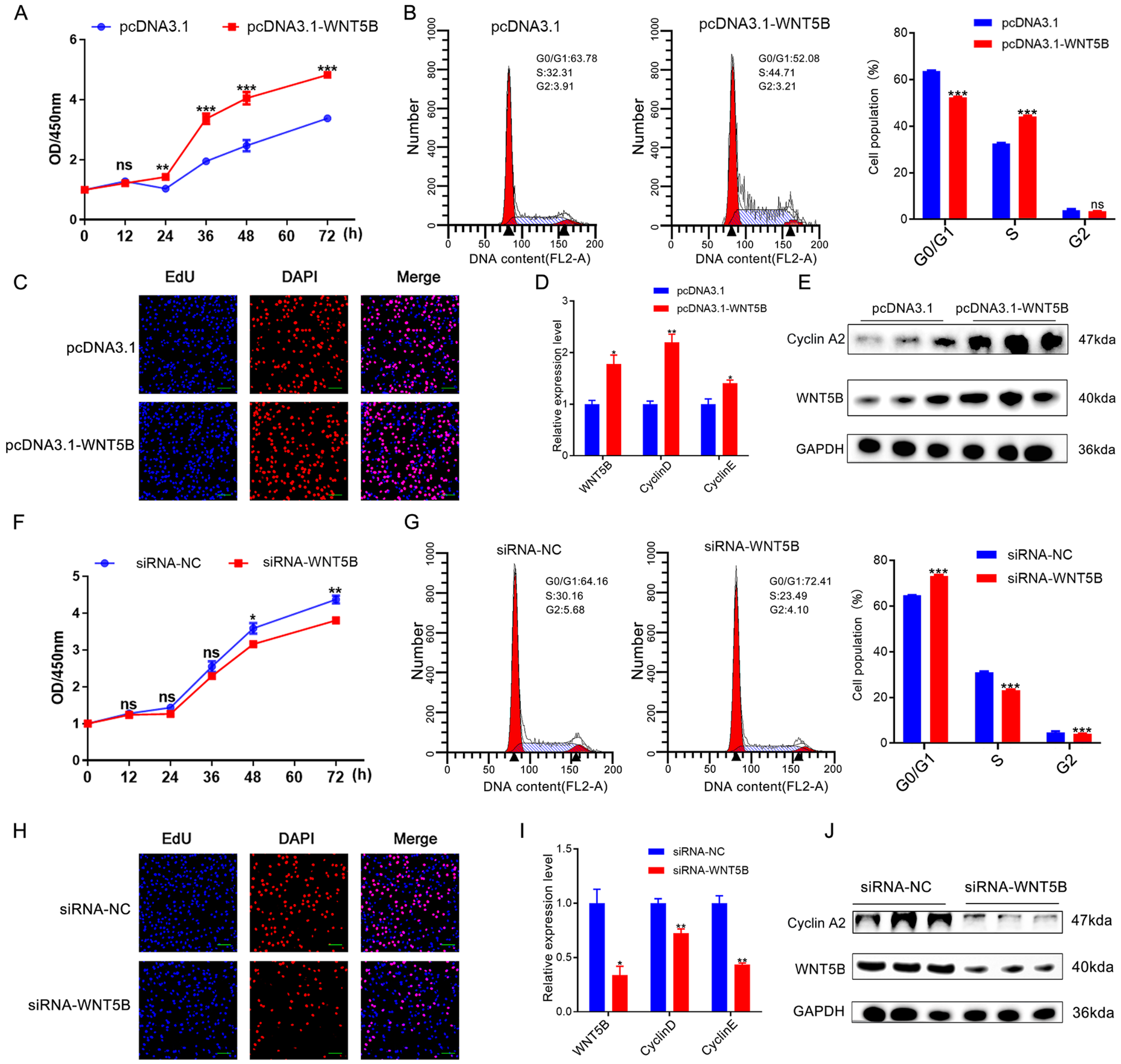

Figure S3. The effects of *WNT5B* on the cell proliferation and cell cycle of C2C12 myoblasts *in vitro*

(A-C) The results of CCK-8 (A), cell cycle (B) and cell proliferation status (C) of C2C12 myoblasts after transfection with pcDNA3.1 and pcDNA3.1-*WNT5B* vectors. Scale bar, 50 μm.

(D-E) mRNA (D) and protein (E) expression levels of proliferation marker genes in C2C12 myoblasts after *WNT5B* overexpression.

(F-H) The results of CCK-8 (F), cell cycle (G) and cell proliferation status (H) of in C2C12 myoblasts after transfection with siRNA-NC and siRNA-*WNT5B*. Scale bar, 50 μm.

(I-J) mRNA (I) and protein (J) expression levels of proliferation marker genes in C2C12 myoblasts after *WNT5B* knockdown.

Data are presented as mean ± SEM and analyzed for statistical differences between groups using unpaired two-tailed t-tests. *$p < 0.05$, **$p < 0.01$, ***$p < 0.001$, ns means no significant differences.

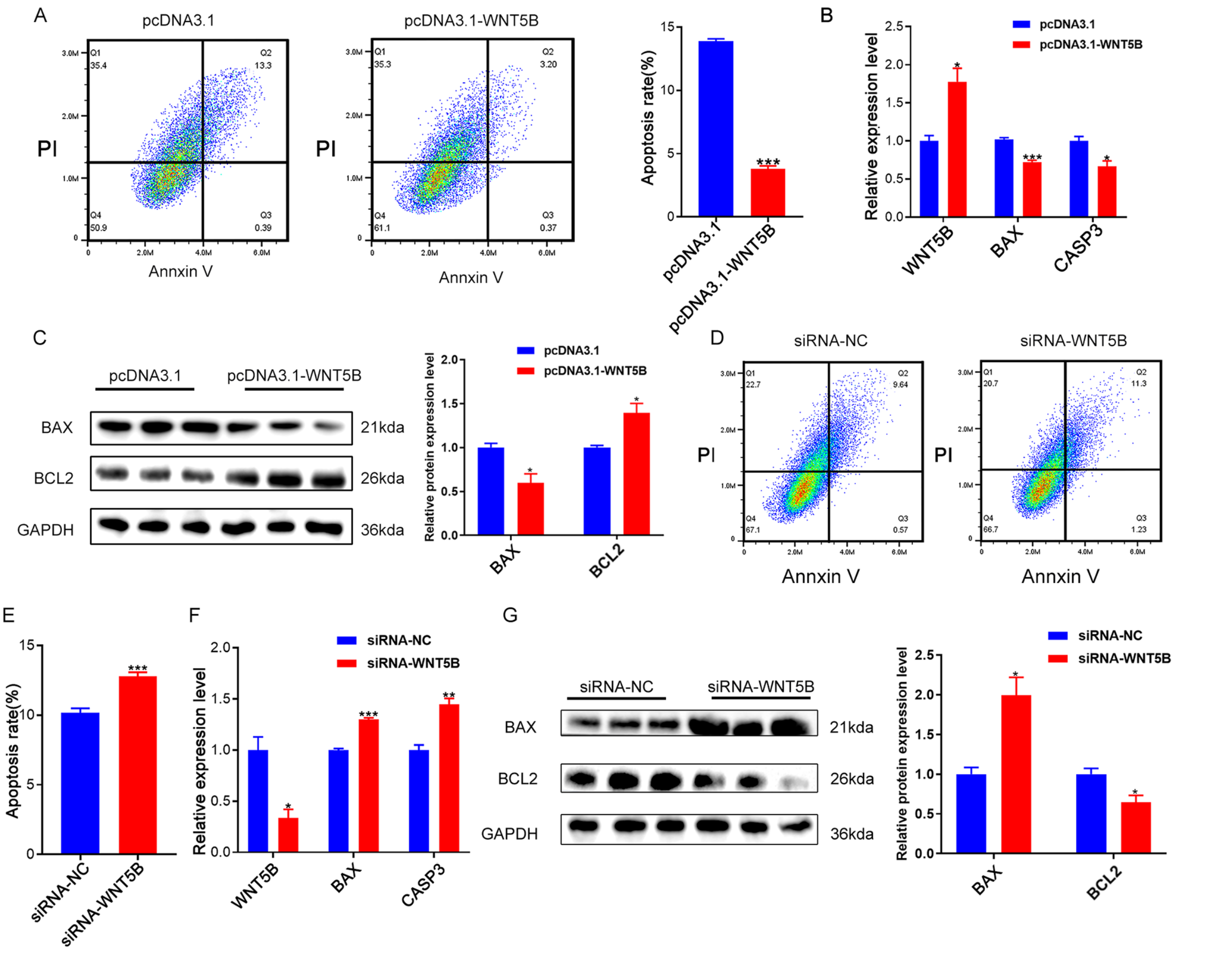

Figure S4. ***In vitro*** **experiments on the effects of** ***WNT5B*** **on cell apoptosis in C2C12 myoblasts**

(A) The results of cell apoptosis of C2C12 myoblasts after *WNT5B* overexpression.

(B-C) mRNA (B) and protein (C) expression levels of cell apoptosis markers genes in C2C12 myoblasts after overexpression of *WNT5B*.

(D-E) The results of cell apoptosis of C2C12 myoblasts after *WNT5B* knockdown.

(F-G) mRNA (F) and protein (G) expression levels of cell apoptosis markers genes in C2C12 myoblasts after knockdown of *WNT5B*.

Data are presented as mean ± SEM and analyzed for statistical differences between groups using unpaired two-tailed t-tests. $*p < 0.05$, $**p < 0.01$, $***p < 0.001$, ns means no significant differences.

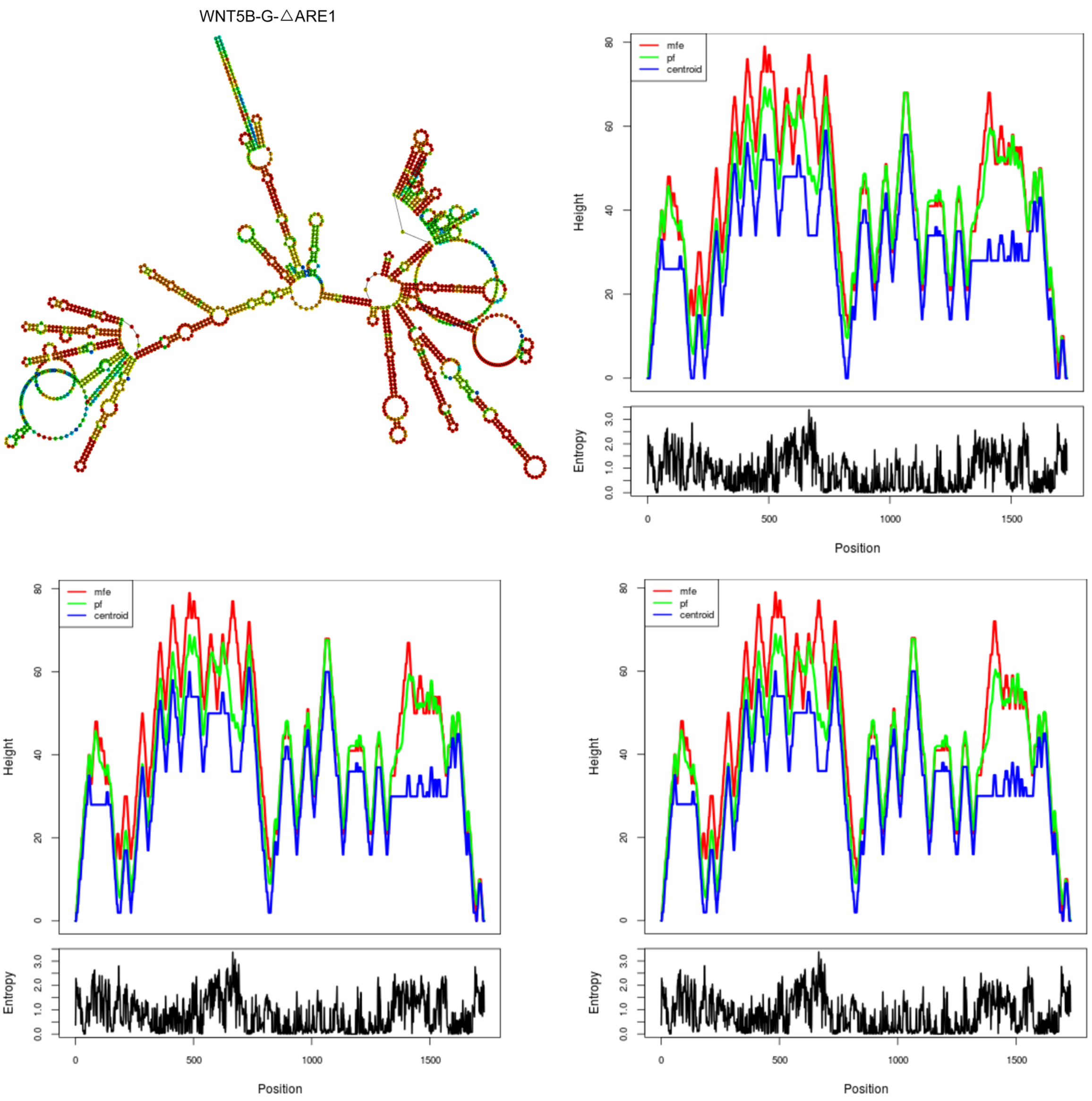

Figure S5. RNAfold tool to analyze the effects of △ARE1 and △ARE2 on *WNT5B* mRNA structure

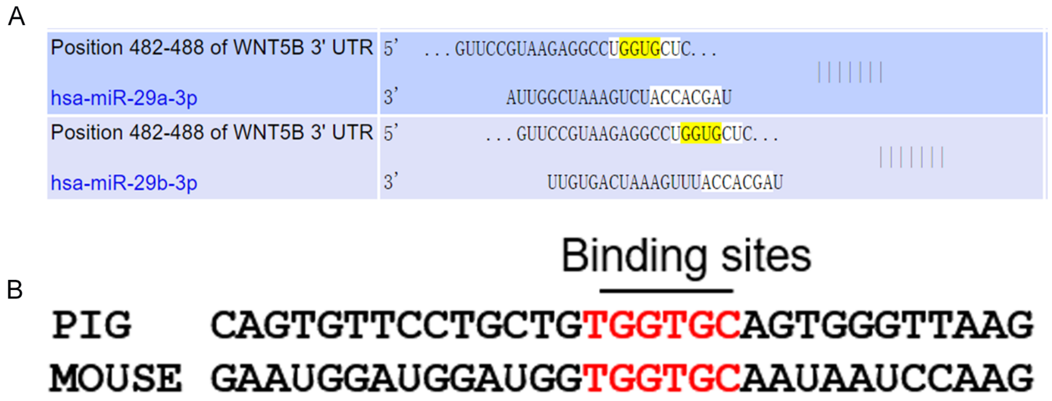

Figure S6. Analysis of *WNT5B* 3'UTR sequences

(A) Prediction of binding sites between the miR-29a/b/c and *WNT5B* using Target Scan.

(B) Amplification of miR-29a/b/c binding sites in the *WNT5B* 3'UTR.

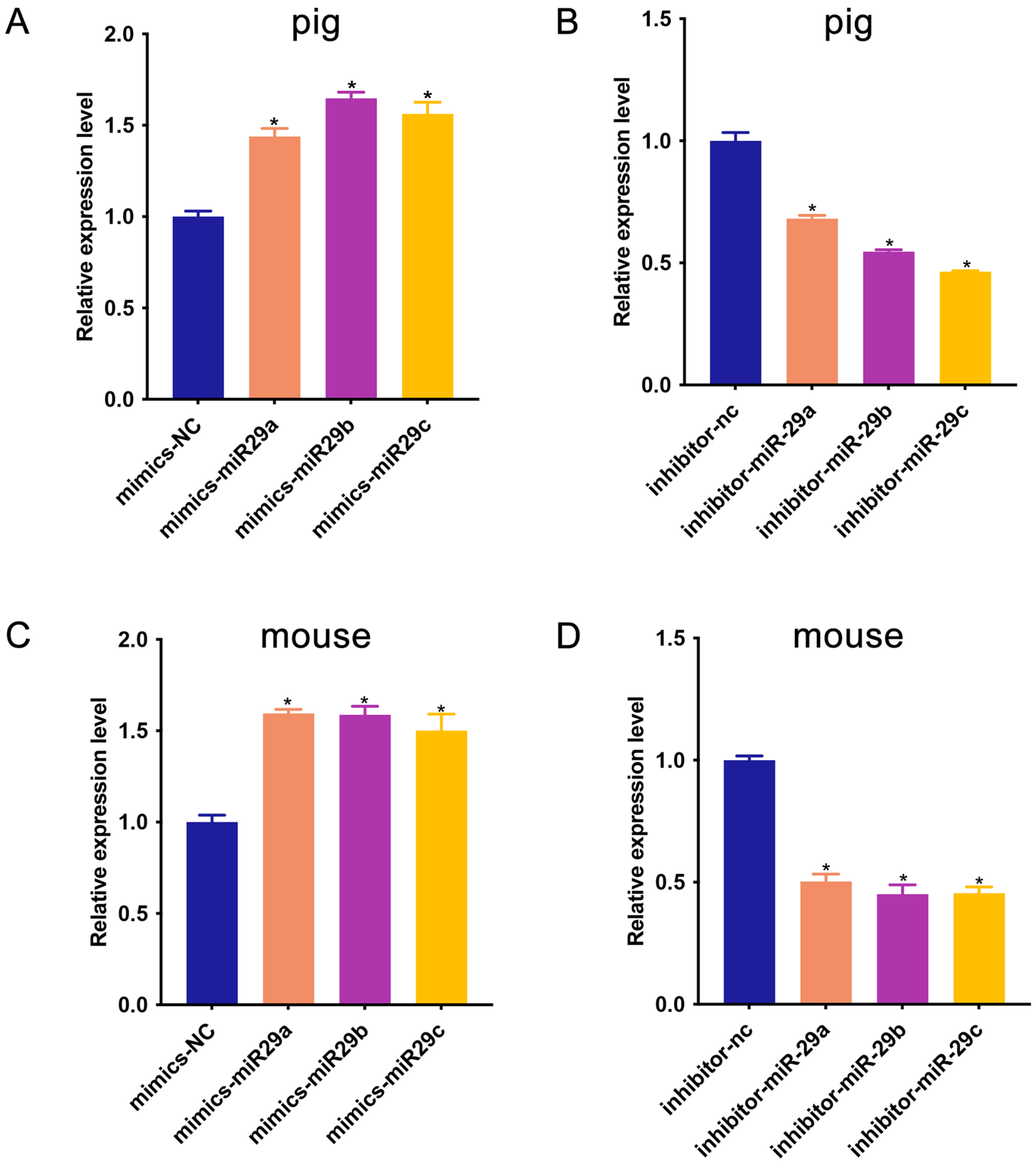

Figure S7. The efficiency of overexpression and knockdown of miR-29/b/c.

(A-B) The efficiency of overexpression (A) and knockdown (B) of miR-29/b/c in porcine myoblasts.

(C-D) The efficiency of overexpression (C) and knockdown (D) of miR-29/b/c in C2C12 myoblasts.

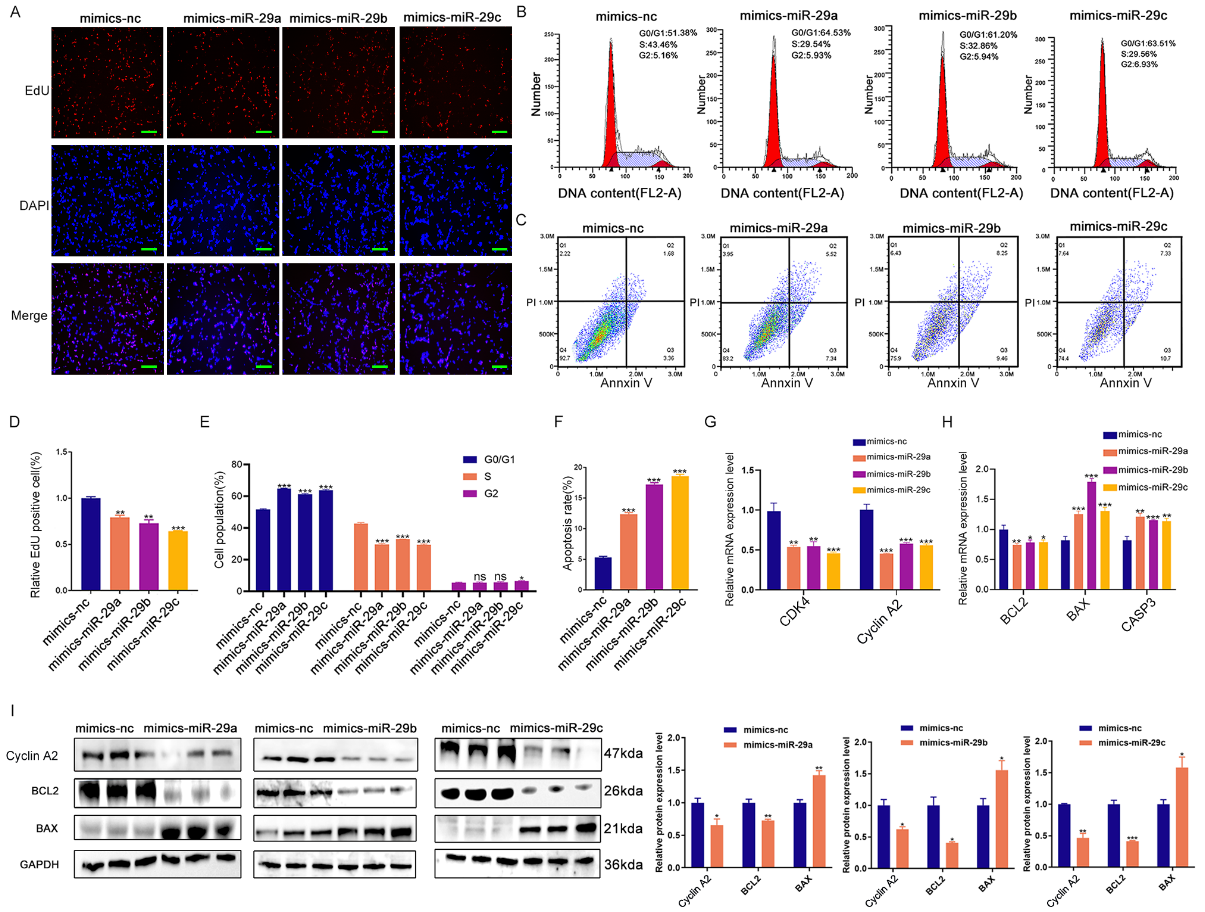

Figure S8. The effects of miR-29a/b/c overexpression on cell proliferation, cell cycle and cell apoptosis in porcine myoblasts

(A-C) The results of cell proliferation (A), cell cycle (B), and cell apoptosis (C) after transfection with miR-29a/b/c mimics in porcine skeletal muscle cells. Scale bar, 50 μm.

(D-F) Quantitative results of cell proliferation (D), cell cycle (E), and cell apoptosis (F).

(G-H) The mRNA expression of cell cycle (G) and cell apoptosis (H) markers expression after miR-29a/b/c overexpression in porcine skeletal muscle cells.

(I) The protein expression of cell cycle and cell apoptosis markers after miR-29a/b/c overexpression in porcine skeletal muscle cells.

Data are presented as mean ± SEM and analyzed for statistical differences between groups using unpaired two-tailed t-tests. *$p < 0.05$, **$p < 0.01$, ***$p < 0.001$, ns means no significant differences.

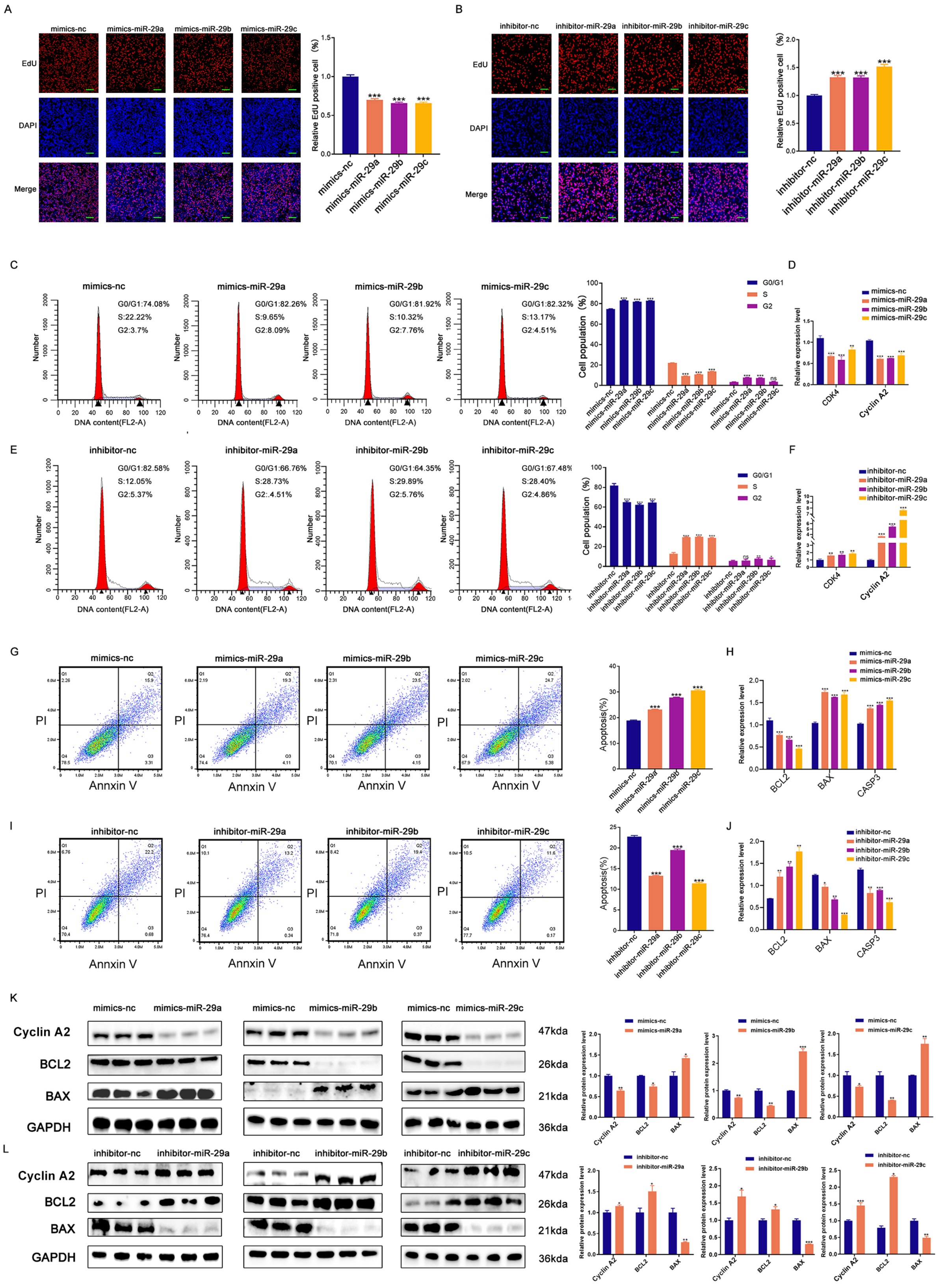

Figure S9. The effects of miR-29a/b/c on cell proliferation, cell cycle and cell apoptosis in C2C12 myoblasts

(A-B) The results of EdU-staining in cell proliferation after miR-29a/b/c overexpression (A) and knockdown (B) in C2C12 myoblasts. Scale bar, 50 µm.

(C-D) Cell cycle results (C) and the mRNA (D) expression of cell proliferation marker gene after miR-29a/b/c overexpression in C2C12 myoblasts.

(E-F) Cell cycle results (E) and the mRNA (F) expression of cell proliferation marker gene after miR-29a/b/c knockdown in C2C12 myoblasts.

(G-H) Cell apoptosis results (G) and the mRNA (H) expression of cell apoptosis marker gene after miR-29a/b/c overexpression in C2C12 myoblasts.

(I-J) Cell apoptosis results (E) and the mRNA (F) expression of cell apoptosis marker gene after miR-29a/b/c knockdown in C2C12 myoblasts.

(K-L) The protein expression of Cyclin A2, BAX, and BCL2 after miR-29a/b/c overexpression (K) and knockdown (L) in C2C12 myoblasts.

Data are presented as mean ± SEM and analyzed for statistical differences between groups using unpaired two-tailed t-tests. *$p < 0.05$, **$p < 0.01$, ***$p < 0.001$, ns means no significant differences.

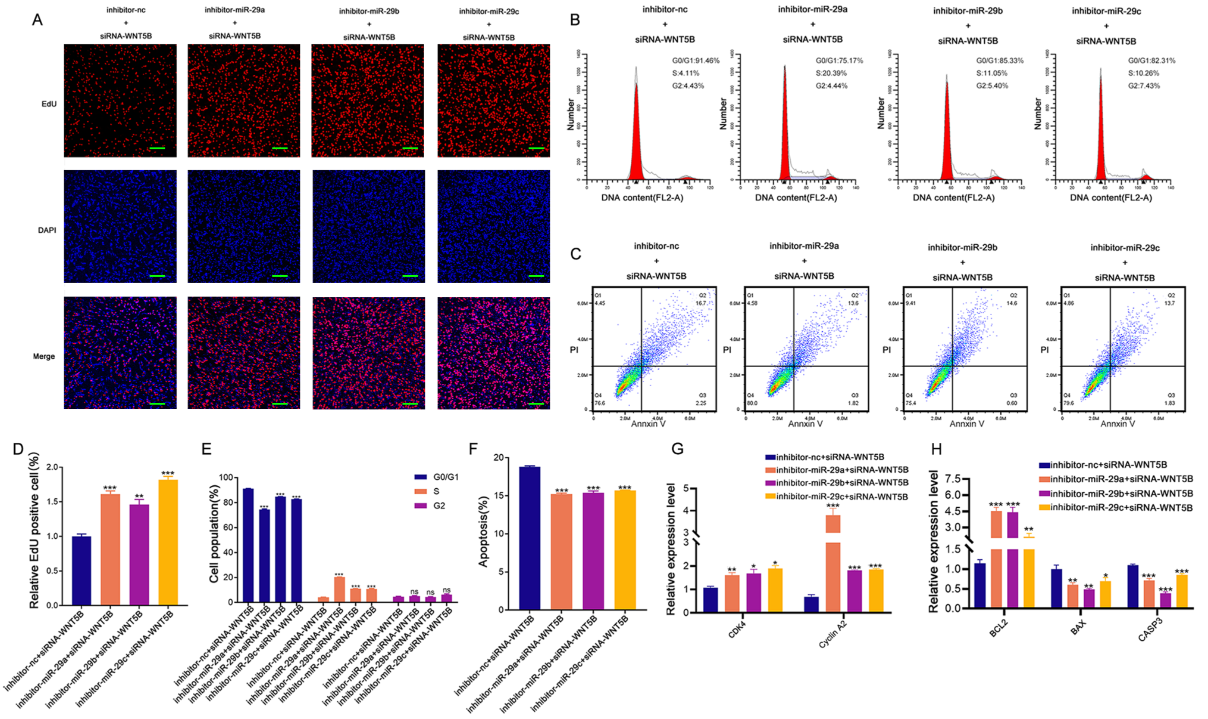

Figure S10. miR29 family targeting *WNT5B* regulates C2C12 myoblast proliferation, cell cycle, and cell apoptosis

(A-C) The results of cell proliferation (A), cell cycle (B), and cell apoptosis (C) after co-transfection with miR-29a/b/c inhibitor and *WNT5B* siRNA in C2C12 myoblast. Scale bar, 50 µm.

(D-F) Quantitative results of cell proliferation (D), cell cycle (E), and cell apoptosis (F).

(G-H) The expression of cell cycle (G) and cell apoptosis markers (H) in mRNA level after co-transfection with miR-29a/b/c inhibitor and *WNT5B* siRNA in C2C12 myoblast.

Data are presented as mean ± SEM and analyzed for statistical differences between groups using unpaired two-tailed t-tests. *$p < 0.05$, **$p < 0.01$, ***$p < 0.001$, ns means no significant differences.

Supplementary Tables

Table S1. Small RNA was used in this manuscript

Name	Target sequence（5'-3')
miR-29c-mimics	UAGCACCAUUUGAAAUCGGUUA
miR-29c-inhibitor	UAACCGAUUUCAAAUGGUGCUA
miR-29b-mimics	UAGCACCAUUUGAAAUCAGUGUU
miR-29b-inhibitor	AACACUGAUUUCAAAUGGUGCUA
miR-29a-mimics	CUAGCACCAUCUGAAAUCGGUUA
miR-29a-inhibitor	UAACCGAUUUCAGAUGGUGCUAG
mimics NC	UUGUACUACACAAAAGUACUG
Inhibitor NC	CAGUACUUUUGUGUAGUACAA
mmu-siRNA-NC	GCGACGAUCUGCCUAAGAU
mmu-siRNA-WNT5B	GGGUGAGUUGCACAGUGAAUC UUCACUGUGCAACUCACCCUG
ssc-siRNA-NC	UGCUCAGACUCGUAACUG
ssc-siRNA-WNT5B	GGUGAGUUGCACAGUGAAUCG AUUCACUGUGCAACUCACCCU

Table S2. Primers for *WNT5B* 3'UTR dual luciferase reporter vector construction in this manuscript

Name	Forward primer (5'-3')	Reverse primer (5'-3')
WNT5B-ARE	GCTAGCACCAAGGGATATCCACCATA	TCTAGATACACTGCAGCTCATGGCAACAT
WNT5B-ARE1	CCTTGGCTTTAGTTGCTAGCATGTAACCAATAAACCAGCCAG	GCTAGCAACTAAAGCCAAGGA
WNT5B-ARE2	CTGGGGAACCCAACATGTACTTATATTAGGTGCTCAAAGTGCA	TCTAGATACACTGCAGCTCATGGCAACAT

Table S3. qRT-PCR Primers used in this manuscript

**Name**	**Forward primer (5’-3’)**	**Reverse primer (5’-3’)**
mmu-*WNT5B*	CTGCTGACTGACGCCAACT	CCTGATACAACTGACACAGCTTT
mmu-*CDK4*	GAAGCCAGAGAACATTCTAGTGAC	TCGAGGCCAGTCGTCTTCT
mmu-Cyclin A2	TTACCCGGAGCAAGAAAAC	TCTGGCTGCCTCTTCATG
mmu-Cyclin D	AATGCCAGAGGCGGATGA	AAAATGCCAGAGGCGGATGA
mmu-*BAX*	GTGATGGCATGGGACATAGCTC	TGGCGTAGACCTTGCGGATAA
mmu-*BCL2*	GCAGGCAGCTTGAAAGAAAC	GCTGGCCTTTCATGACTCTC
mmu-*CASP3*	CTGCGGCGGGGAGCT	GGTTGGCTGCGTCCACAT
mmu-*GAPDH*	GGTTGTCTCCTGCGACTTCA	TGGTCCAGGGTTTCTTACTCC
ssc-*WNT5B*	GGTGGTCCTTGGCCATGA	AGGCTACGTCTGCCATCTTATAC
ssc-*CDK4*	GCGTAAGAGTCCCCAATGGA	AGACATCCATCAGCCGGACA
ssc-Cyclin A2	TCTATGGCGGAAGTTCTTGCT	CACTGCCCATGCTGGTAGAA
ssc-*BAX*	GCCCTTTTGCTTCAGGGTTTC	GCCCTTTTGCTTCAGGGTTTC
ssc-*BCL2*	GGATAACGGAGGCTGGGATG	TTATGGCCCAGATAGGCACC
ssc-*CASP3*	CTGGCGAAATTCAAAGGAC	AACCATTTCCTCATTTCACATAC
ssc-*MYHC*	GTTCAGAGAAAGGCATCCCAAA	GAGAGTGACCGACACCACAAGTG
ssc-*MYH7*	AAGGGCTTGAACGAGGAGTAGA	TTATTCTGCTTCCTCCAAAGGG
ssc-*MYH4*	ATGAAGAGGAACCACATTA	TTATTGCCTCAGTAGCTTG
ssc-*TNNI1*	CCCACAGTCTGCAGTCCAC	CCAGCATCAGGCCCTTCAG
ssc-*TNNI2*	TCCAGGAGCTCTGCAAACAG	GGTTCATGTCCTCCAGCTCC
ssc-*TNNT1*	CCAAGCCAAGCCGTCCC	CAATACGCTCTTTCAGCGCC
ssc-*TNNT3*	CATCATCGCCAAGGGTTCTTTCA	TGCCTGGATGGTAGTAGAGCA
ssc-*NEAT1*	GTCGATGCCCTGAACATG	GTCGATGCCCTGAACATG
ssc-*GAPDH*	TTATGGCCCAGATAGGCACC	TTATGGCCCAGATAGGCACC
miR-29a	GCGCTAGCACCATCTGAAAT	AGTGCAGGGTCCGAGGTATT
miR-29b	CGCGTAGCACCATTTGAAATC	AGTGCAGGGTCCGAGGTATT
miR-29c	CGCGTAGCACCATTTGAAAT	AGTGCAGGGTCCGAGGTATT
